# Do ESG Frameworks Capture Corporate Health Impacts? An Analysis of the Food and Beverage Industry

**DOI:** 10.3390/ijerph23010030

**Published:** 2025-12-24

**Authors:** Raquel Burgess, Kenneth Chen, Savas (Jitae) Kim, Naisha Dharia, Christine Lin, Tanja Srebotnjak, Lawrence Grierson, Nicholas Freudenberg, Daniel C. Esty, Yusuf Ransome

**Affiliations:** 1 Department of Social and Behavioral Sciences, Yale School of Public Health, Yale University, New Haven, CT 06510, USA; 2 Munk School of Global Affairs and Public Policy, University of Toronto, Toronto, ON M5S 0A7, Canada; kenn.chen@mail.utoronto.ca; 3 Honours Health Sciences Program, McMaster University, Hamilton, ON L8S 4L8, Canada; 4 Zilkha Center for the Environment, Williams College, Williamstown, MA 01267, USA; 5 Department of Family Medicine, McMaster University, Hamilton, ON L8P 1H6, Canada; 6 Department of Community Health and Social Sciences, CUNY Graduate School of Public Health and Health Policy, City University of New York, New York City, NY 10027, USA; 7 Yale School of the Environment, Yale University, New Haven, CT 06511, USA; 8 Yale Law School, Yale University, New Haven, CT 06511, USA

**Keywords:** commercial determinants of health, investment, environmental social governance, ESG, non-financial reporting, food and beverage, sustainability, health, corporate

## Abstract

Investors use information about companies’ social and environmental performance to make investment decisions, a strategy known as Environmental, Social, and Governance (ESG) investment analysis. ESG screening may offer a mechanism to incentivize corporations to improve their health impact. However, there has been limited investigation of the extent to which ESG investment frameworks capture corporate health impacts in major industries. In this study, we sought to characterize the extent to which ESG frameworks address the health-impacting activities of the food and beverage (F&B) industry. To do this, we conducted a deductive framework analysis during the period of September 2023 to March 2024. Specifically, we identified gaps in existing ESG frameworks by comparing the content of five ESG reporting standards and rating systems to the HEALTH-CORP-FB typology, an evidence-based typology that describes the health-impacting activities of the F&B industry across seven domains (Governance Practices, Political Practices, Preference and Perception Shaping Practices, Economic Practices, Employment Practices, Products and Services, and Environmental Practices). To further assess how ESG frameworks account for the health-impacting activities of the F&B industry, we classified health-focused ESG fields in the packaged foods subindustry by two attributes: relevance to the assigned HEALTH-CORP-FB activity (low, medium, high) and type of business operations addressed (e.g., process, performance). Results indicate that, on average, the ESG fields (*n* = 1348) covered 39% of the 89 HEALTH-CORP-FB activities (range across frameworks: 27–48%). Higher proportions of activities in the Governance, Environmental, Employment, and Economic Practices domains (range across domains: 43–87%) were represented than activities in the Products and Services, Preference and Perception-Shaping Practices, and Political Practices domains (17–36%). Fields assigned to the latter domains were also less likely to be deemed highly relevant and to measure corporate performance. We conclude that the ESG frameworks included in this study capture some of the activities of the F&B industry that affect population health and health equity; however, critical gaps remain. We discuss how integrating key health-focused ESG indicators (e.g., revenue generation from ultra-processed foods) into existing frameworks could enable investors, public health organizations, civil society, and shareholder advocates to strengthen the accountability of the F&B sector with respect to health.

## 1. Introduction

Public health evidence demonstrates that the activities of commercial entities have measurable impacts on population health [1,2,3,4]. For example, just four products (i.e., tobacco, alcohol, fossil fuels, and ultra-processed foods) are estimated to account for one-third of annual preventable deaths globally [1]. The profits from these products flow to companies and their shareholders, while the resulting health and social costs—such as disease burden, premature mortality, and healthcare expenditures—are borne by society [1]. These dynamics exemplify negative externalities, where the true costs of harmful products are not reflected in their market price [1].

The impact of commercial entities on population health and the economic and political structures, norms, and values that facilitate these impacts is increasingly studied through the lens of the commercial determinants of health (CDOH). Research and global interest in the CDOH have accelerated in recent years [1,5,6,7,8] because of concerns about the political and economic power wielded by large, transnational corporations and recognition of their significant impacts on population health [9].

Despite substantial advances in the field, CDOH researchers note that two critical priorities have yet to be fully addressed. The first priority requires developing systems to measure and monitor the practices of commercial entities that influence population health [6,9,10,11,12,13,14]. The second priority entails identifying and leveraging mechanisms to discourage commercial practices that harm human health and encourage those that promote health [5,9,13,15,16,17,18,19,20].

Environmental, Social, and Governance (ESG) investment analysis offers a potential mechanism to advance these priorities. Investors who engage in ESG screening use information about how a company manages social and environmental risks and opportunities to inform their investment decisions, alongside traditional financial analysis [21,22].

The three ESG pillars are corporate Environmental performance (e.g., greenhouse gas emissions), Social performance (e.g., payment of fair wages), and the effectiveness by which the company is managed (i.e., Governance (e.g., corruption prevention mechanisms)) [23]. Investors typically use ESG data to align their investments with their core values and beliefs and/or to gain a financial advantage [24,25,26]. The latter strategy derives from the (contested) belief that companies that better manage environmental and social risks and capitalize on environmental and social opportunities will deliver superior shareholder returns than companies that do not [22,27,28,29,30,31,32].

ESG screening originates in the actions of the United Nations. In the 1990s, the United Nations shifted from an oppositional to a collaborative approach with the private sector, leading to the launch of the UN Global Compact in 2000 [33,34]. This voluntary initiative encourages corporations to uphold standards related to human rights, labor, environment, and anti-corruption [34]. It has now been embraced by over 20,000 signatories in more than 160 countries [35]. The Global Compact was followed by two additional UN-affiliated reports, “Who Cares Wins” [36] and the Freshfields report [37], which suggested that companies with strong ESG performance will financially outperform their counterparts and that, because of this, it is legally permissible for investment managers to use ESG factors in their investment decisions within major jurisdictions (e.g., the United Kingdom, the United States). Building on these efforts, the UN established the Principles for Responsible Investment (PRI) in 2006, which encourages investors to integrate ESG issues into investment decisions [38].

Currently, ESG investment analysis is operationalized through a series of reporting standards and ratings providers/data aggregators [39,40]. Reporting standards provide guidelines for the information that companies disclose in ESG reports (also referred to as non-financial reports and sustainability reports) [41,42]. Ratings providers and data aggregators are third-party organizations that use corporate disclosures, questionnaires issued to corporations, and/or other publicly accessible data to generate ESG ratings, rankings, and/or ESG datasets [43]. Those that manage funds on behalf of others (i.e., money managers, such as pension fund managers) may then decide to use these ESG products to inform their investment decisions [43].

In 2024, Bloomberg Intelligence estimated that ~29% of global projected assets under management ($40 trillion USD) will be managed using ESG criteria by 2030 [44]. Despite its popularity, however, there is also significant controversy surrounding ESG screening, particularly in the United States. This controversy stems from the belief that investment managers engaging in ESG screening are injecting their own values into investment decision making and violating their fiduciary duty to maximize returns for shareholders [45]. In addition to this conceptual critique, the varying uses of ESG information (i.e., for moral and financial purposes) and ongoing debate about the financial relevance of ESG information have generated significant confusion about its nature and purpose [24,27,45]. Finally, ESG data is of highly variable quality, not sufficiently comparable across companies, vulnerable to “greenwashing”, and the data are often not adequately assured (i.e., verified for accuracy) [46,47,48,49]. Influential bodies such as the International Sustainability Standards Board (ISSB) and the European Commission [50,51,52,53,54] have recently engaged in efforts to ameliorate these issues and increase investor confidence in ESG data. Meanwhile, the current Trump administration’s deregulatory, pro-business, and anti-climate agenda has led to significant outflows from ESG funds by US investors and a wave of “anti-ESG legislation” in Republican-led states [55].

Against this backdrop, there has been recent interest in ESG screening as an avenue to incentivize corporations to improve their impact on nutrition and human health [5,56,57,58]. The potential benefits of ensuring that corporate health impacts are considered within ESG investment strategies are two-fold. First, if a significant proportion of assets are managed in ways that consider companies’ health impacts, companies may be incentivized to improve these impacts to retain and attract investment capital [27,59,60,61,62]. Second, existing and future ESG reports could become a source of publicly accessible data points to monitor commercial practices that affect population health.

Despite the focus of ESG criteria on “social impact”, initial evidence suggests that corporate health impacts are not adequately accounted for within existing ESG frameworks [57,63,64,65,66]. For example, O’Hearn and colleagues conducted an evaluation of the strength of ESG nutrition-focused metrics (e.g., revenue from zero-calorie and low-calorie, no-added sugar, and artificially sweetened beverages) from eight ESG frameworks. They found that most metrics focused on product marketing, with limited metrics capturing product healthiness or the distribution of F&B products and subsequent impacts on health equity [57].

In this study, we expanded on this work by investigating the extent to which five existing ESG frameworks capture the activities of the food and beverage (F&B) industry across several dimensions (e.g., nutrition, environmental, employment, political). Like O’Hearn and colleagues [57], we decided to focus on the F&B industry because of its substantial impact on human health [67,68,69,70,71]. However, we sought to broaden the focus beyond nutrition metrics to also evaluate the extent to which ESG frameworks capture the F&B industry’s health impacts through means such as employee working conditions, contributions to climate change, efforts to shape food desires and preferences, and the industry’s deep influence on the implementation of policies designed to promote healthier eating patterns [67,68,69,70,71].

Our findings suggest that the ESG frameworks included in this study capture some of the activities of the F&B industry that affect population health and health equity (e.g., occupational injuries, non-compliance with marketing practices). However, critical gaps exist in the coverage of some types of activities, such as their political practices (e.g., litigation practices, lobbying activities). We leverage these findings to offer recommendations for strengthening existing ESG frameworks to improve the accountability of the F&B industry. We also discuss the implications of the findings for ESG framework producers, F&B companies, policy makers, and the public health community.

## 2. Methods

The primary research question guiding this study was: To what extent do existing ESG frameworks capture the activities through which the F&B industry influences health? While earlier research has primarily examined nutritional aspects, our focus was broader—evaluating whether ESG frameworks account for health influences across multiple dimensions, including nutrition, environmental factors, employment practices, and political activities. Our secondary research question was: If health impacts of the F&B industry are addressed within existing ESG frameworks, how are these impacts captured?

To address the primary research question, we conducted a deductive framework analysis to compare the content of five existing ESG frameworks to a previously developed typology that describes the activities of the F&B industry that can influence population health and health equity (called the Corporate Influences on Population Health—Food and Beverage (HEALTH-CORP-FB) typology) [72,73]. The HEALTH-CORP-FB typology is an evidence-based framework that describes 89 activities through which the F&B industry can influence population health (e.g., product marketing, employee working conditions). These activities are categorized across seven domains of corporate influence (e.g., Political Practices, Employment Practices) [72] (see Tables S2 and S3 for the full typology). Our aim was to compare the content of existing ESG frameworks to the HEALTH-CORP-FB typology to identify which HEALTH-CORP-FB activities are already captured within the ESG frameworks and which are not. In order to do this, we employed deductive framework analysis, a qualitative approach to analyzing data by comparing it to a pre-existing theoretical or conceptual framework [74,75]. This method allowed us to systematically and consistently compare the content of the ESG frameworks to that of the HEALTH-CORP-FB typology.

Where we found indicators in the ESG frameworks that captured activities in the HEALTH-CORP-FB typology, we further documented the type of business operations these indicators measured (e.g., commitments, processes, performance) and their relevance to the respective HEALTH-CORP-FB activity (i.e., low, medium, or high). This allowed us to glean insights about how existing ESG frameworks capture the impact of F&B companies on human health (our secondary research question).

**Figure 1:**
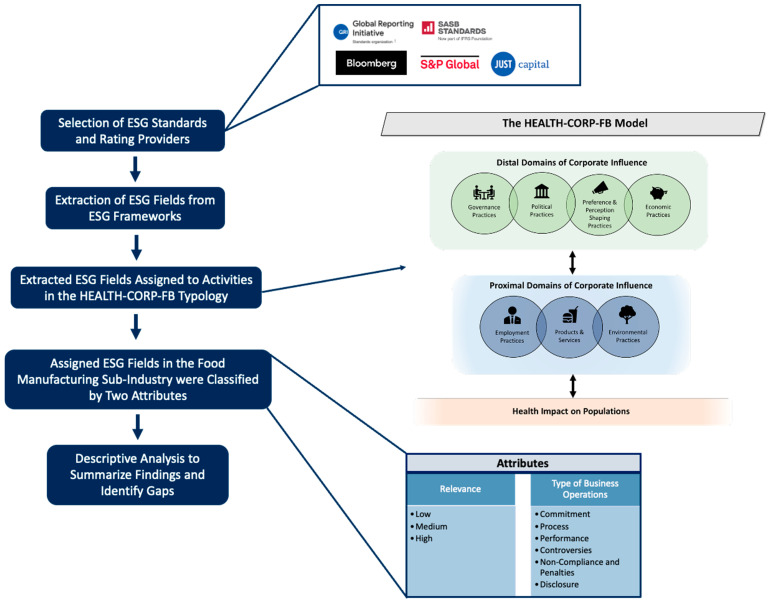
Graphical overview of the study procedures. For more information about the HEALTH-CORP-FB Model and Typology and its development, please consult our published work [72].

Our methods are adapted from those developed by O’Hearn and colleagues to evaluate the strength of ESG-Nutrition metrics [57]. A graphical overview of the study procedures is provided in Figure 1 and further described below. This study was not submitted to an institutional review board as it did not involve human participants.

### 2.1. Selection of ESG Standards and Ratings Providers

We selected two ESG reporting standards and three ESG ratings systems for inclusion in the study (Table 1). The ESG reporting standards (Sustainability Accounting Standards Board (SASB) [76] Standards, Global Reporting Initiative (GRI) Standards) [77] were purposively chosen for inclusion based on the large number of companies globally that use these standards to report non-financial information and their use by major F&B companies (e.g., PepsiCo, Inc., Néstle S.A.) [78]. The GRI and SASB Standards also reflect two different approaches to ESG screening. The SASB Standards ask companies to report ESG information that is deemed financially relevant [76]. This is often referred to as a financial materiality approach to ESG screening [79]. The GRI Standards, however, encourage companies to report on their impacts on people, people, the planet, and the economy, regardless of the short-term financial implications of these activities [80]. This is known as an impact materiality approach [79]. Including both the GRI and SASB Standards in our study allowed for a more comprehensive assessment of how health impacts of the F&B industry are captured across both impact- and financial-oriented ESG frameworks. Importantly, the SASB and GRI Standards also play essential roles in the influential International Financial Reporting Standards (IFRS) Sustainability Disclosure Standards recently published by the International Sustainability Standards Board (ISSB) and the European Sustainability Reporting Standards (ESRS) adopted by the EU, respectively [50,52,81].

The three ESG ratings providers were selected based on a purposive and convenience sampling strategy. These providers are widely recognized in the ESG investment and corporate responsibility landscape [43,82]. Each employs distinct definitions of materiality and unique evaluation methodologies, allowing for a varied sample (Table 1). Additionally, their methodologies were either publicly available or accessible through our existing institutional subscriptions. Due to high access costs, we were not able to include other widely used ESG data sources such as Sustainalytics or MSCI. For the remainder of this article, we collectively refer to the ESG standards and ratings systems as ESG frameworks.

### 2.2. ESG Field Extraction

We accessed the most recent version of the ESG frameworks from the respective websites and portals. Next, we extracted the respective ESG fields (e.g., “(1) Total recordable incident rate (TRIR) and (2) fatality rate”, SASB Standards) between September 2023 and March 2024 (see Table S1 for document version dates). We included the methodology for the 2021 S&P Global ESG Scores in our sample because this was the publicly available version at the time of data collection.

For non-industry-specific frameworks (i.e., GRI Standards, JUST Capital Rankings, Bloomberg Governance (G) Scores), we extracted all ESG fields present in the framework. For industry-specific frameworks (i.e., SASB Standards, Bloomberg Environmental Social (ES) Scores, and S&P Global ESG Scores), we extracted fields for all sub-industries relevant to the F&B industry (e.g., agriculture, food retailing), excluding alcoholic beverages because of their unique health impact (Table 1). For the ESG standards, the extracted fields are the disclosures that companies are requested to report within their non-financial reports. For the ratings providers, the extracted fields are the metrics that the provider uses to measure companies’ ESG performance. We use the term ESG fields to collectively refer to disclosures and metrics in the ESG frameworks.

**Table 1:** Characteristics of the ESG frameworks included in this study.

ESG Framework	Type of ESG Framework	Industry-Specific?	Brief Description	Materiality Orientation ^a^	Audience	Coverage	Relevant Food and Beverage Sub-Industries
Global Reporting Initiative (GRI) Standards [Established in 1997]	Reporting Standard	No	The GRI Standards consist of a series of inter-connected standards designed to assist organizations in reporting information about their impacts on the economy, environment, and people. All companies reporting based on the GRI Standards are requested to apply the universal standards (GRI 1, 2, and 3). Companies then use the results of their materiality assessment (i.e., identification of topics leading to significant impacts) to determine which topic-level GRI disclosures they will report (e.g., biodiversity, customer health and safety) [80]. ^b^	Impact materiality: Impacts on the environment, people, and the economy	Businesses, investors, policymakers, civil society, and other stakeholders	~14,000 organizations in over 100 countries use the GRI Standards to report non-financial information [83]	N/A^c^
Sustainability Accounting Standards Board (SASB) Standards [Established in 2011]	Reporting Standard	Yes	The SASB Standards offer industry-specific standards that are designed to aid companies in reporting sustainability-related information deemed relevant to investor decision-making. Companies select the applicable industry(ies), choose which disclosure topics are relevant to their business, and report information based on the Standards that have been developed for that specific industry(ies) [76].	Financial materiality	Primarily investors	3551 companies (170 countries) since 2022 used the SASB Standards to report non-financial information [84] (~196 + F&B companies)	Agricultural Products; Food Retailers and Distributors; Meat, Poultry, and Dairy; Non-Alcoholic Beverages, Processed Foods; Restaurants
Bloomberg ESG Scores [Launched in 2020]	Ratings Provider	Yes ^d^	Bloomberg ESG Scores measure companies’ exposure to and management of industry-specific financially material ESG topics [85]. They are designed for use by institutional investors that wish to use financially material ESG information to inform their investment decisions. ESG Scores are constructed based on publicly available, company-disclosed data.	Financial materiality	Primarily investors	~15,000 companies assessed [86] (~293 F&B companies)	Packaged Food and Beverages (Non-Alcoholic Beverages, Packaged Food); Consumer Discretionary (Restaurants); Retail and Wholesale (Retail, Wholesale); Agriculture (Agricultural Producers and Agricultural Product Wholesalers)
JUST Capital Rankings [Launched in 2016]	Rankings Provider	No ^e^	JUST Capital provides rankings that evaluate companies based on the issues that are prioritized by the American public, determined via a representative survey of the adult population in the United States [87]. They are designed to inform the public about and investors about the performance of large US companies and to incentivize positive corporate change. The rankings are constructed based on publicly available company documents, crowdsourced data, data from government, academic, and nonprofit organizations, RepRisk data, and in-house surveys. Top-performing companies are included in financial products (e.g., the JUST 100 Index) [88].	Issues prioritized by the American public	Companies, investors, the public	Roughly equivalent to the Russell 1000 index (i.e., the 1000 largest, publicly traded U.S. companies) [87] (~77 F&B companies)	N/A^c^
S&P Global ESG Scores [Launched in 2020]	Ratings Provider	Yes	The ESG Scores offered by S&P Global measure corporate management and performance related to material ESG risks. They are designed to inform investors about companies’ ESG performance. The Scores also determine companies’ inclusion in the Dow Jones Sustainability Index [89]. S&P Global constructs the ESG Scores based on corporate responses to the industry-specific Corporate Sustainability Assessment (CSA). ^f^ Scores are also based on controversy data provided by RepRisk.	Double materiality: material issues are those that lead to significant impacts on society or the environment AND have an impact on the company’s financial performance [90]. ^g^	Primarily investors	~13,000 companies as of 2024 [89] (865 F&B companies)^f^	Food and Staples Retailing (FDR), Food Products (FOA), Restaurants and Leisure Facilities (REX), Beverages (BVG) ^h^

^a^ Financial materiality refers to information that is relevant to investor decision-making because it is likely to have an impact on the financial performance of the company. Impact materiality refers to information about the company’s impacts on people and the planet that is relevant to stakeholders. A double materiality lens refers to the consideration of information that is financially relevant and/or relates to the company’s impacts on society [79]. ^b^ As a result, F&B companies may not report on all of the topic-level disclosures we extracted from the GRI Standards. ^c^ The GRI Standards and the JUST Capital Rankings are not industry specific. ^d^ Bloomberg ESG scores comprise Environment Social (ES) scores and Governance (G) scores. The ES scores are industry-specific, whereas the G score methodology applies across all industries. ^e^ JUST Capital produces industry-level rankings as well as overall rankings. The industry-level ranks are based on the overall ranking the company receives. ^f^ As of 2024, ~13,000 companies were invited to participate in the CSA. A score may be computed for companies that decline based on publicly available information. ^g^ The S&P Global approach to double materiality involves mapping the ESG topics based on both their financial relevance and their impact on stakeholders. Weights for each topic are then applied based on the industry-specific position of the respective topic across these two dimensions [90]. The methodology for the 2021 S&P Global ESG Scores (included in this study) appear to have focused more strongly on financial materiality than subsequent versions [91]. ^h^ The S&P Global Corporate Sustainability Assessment (CSA) questionnaire was not publicly available for the Beverages industry. Therefore, this subindustry is not included in our analysis.

### 2.3. Framework Analysis

As previously stated, we employed a deductive form of framework analysis to investigate the extent to which the activities of the F&B industry that influence population health are captured within the ESG frameworks. Framework analysis offers a qualitative approach to systematically summarize data by indexing (i.e., applying pre-defined codes) and charting (i.e., summarizing by category) the data based on a pre-defined analytical framework [74,75]. In this case, we systematically indexed/assigned the fields extracted from the ESG frameworks (i.e., the data) to relevant activities in the HEALTH-CORP-FB typology (i.e., the pre-defined analytical framework). This allowed us to determine which HEALTH-CORP-FB activities were captured by the five ESG frameworks and which were not.

To develop the HEALTH-CORP-FB typology, we first conducted a scoping review of CDOH literature describing corporate activities that influence health across industries [73]. Using a qualitative synthesis process, we synthesized this literature to develop a model and typology (called HEALTH-CORP) that describes the activities through which corporations influence health across several key domains (e.g., political practices, employment practices) [73]. We then recruited 22 academics and public health professionals with relevant expertise in areas such as nutrition, occupational health, and environmental health, to assist us in adapting the HEALTH-CORP typology to the F&B industry [72]. This process resulted in an industry-specific model and typology (i.e., HEALTH-CORP-FB (Food and Beverage)), that describes 89 activities through which F&B companies influence health (e.g., engaging in excessive product processing, lobbying against public health policies) which are categorized across seven domains (i.e., Governance Practices, Political Practices, Preference and Perception-Shaping Practices, Economic Practices, Employment Practices, Products and Services, and Environmental Practices) [72]. The HEALTH-CORP-FB model is depicted in Figure 1 and the full HEALTH-CORP-FB typology (including the 89 activities categorized across the seven domains) is provided in Table S2.

ESG fields could be assigned to more than one HEALTH-CORP-FB activity if the content pertained to more than one. For example, the ESG field “Number of incidents of non-compliance with industry or regulatory labeling and/or marketing codes” in the SASB Standards was assigned to both “comprehensiveness and accuracy of product nutritional information” and “physical and digital marketing” in the HEALTH-CORP-FB typology. If ESG fields were not relevant to any specific activity but were relevant to an overarching domain, they were assigned to the domain. For example, an ESG field which measured the number and amount of significant fines for environmental violations was mapped to the Environmental Practices domain as opposed to a specific HEALTH-CORP-FB activity. SJK, KC, and ND performed all initial assignments, and RB reviewed all assignments to ensure consistency across frameworks.

### 2.4. ESG Field Classification

Following the framework analysis, we classified the assigned ESG fields by two attributes: relevance and type of business operations (Table 2), which are based on the scoring attributes developed by O’Hearn and colleagues [57]. Relevance refers to the extent to which the ESG field is highly relevant and specific to the HEALTH-CORP-FB activity to which it was assigned (low, medium, or high). Type of business operations refers to the type of business activities the ESG field captures (i.e., commitment, process, performance, controversies, non-compliance and penalties, and disclosure) (see Table 2 for definitions). While the findings of the framework analysis indicated which HEALTH-CORP-FB fields have corresponding ESG fields, further categorizing the fields by relevance allowed us to distinguish between fields that capture the HEALTH-CORP-FB activity in a highly specific and targeted way and those that capture the respective HEALTH-CORP-FB activity in a broader and less targeted manner (see Table 2 for examples). Categorizing the ESG fields by type of business operations (e.g., process, performance) allowed us to identify how health-impacting activities were measured in the ESG frameworks. As recommended by O’Hearn and colleagues, we consider metrics measuring corporate performance (e.g., number of advertising impressions on children) to be more useful than metrics measuring other types of business activities, such as commitments (e.g., commitment to not advertise to children) or controversies (e.g., controversies surrounding marketing practices).

For the industry-specific ESG frameworks, we classified fields in the packaged foods subindustry (as opposed to all F&B-relevant sub-industries). Specifically, we classified fields in the SASB Processed Foods industry, the Bloomberg Packaged Food subindustry, and the S&P Global Food Products subindustry (Table S4 provides definitions). We did this for feasibility purposes and because the industry-specific frameworks used many of the same metrics across F&B sub-industries.

For type of business operations, we classified the ESG fields into more than one attribute category if the field captured more than one category. For example, the ESG field “403–9: Work-related injuries” from the GRI Standards was classified as capturing both performance (i.e., fatalities and work-related injuries) and processes (i.e., actions the company has taken to eliminate work-related hazards).

All classifications for type of business operations were conducted by one coder (i.e., SJK, KC, or RB) and reviewed by a second coder for verification. Any disagreements were resolved via weekly discussions with SJK, KC, RB, and ND. Given that the classifications for relevance required more subjective judgment than the type of business operations, we employed two coders (RB and CL) to conduct the classifications independently. Disagreements were resolved via weekly discussions between RB and CL. The team also met regularly to discuss and refine the operational definitions (Table 2).

### 2.5. Statistical Analyses

We summarized the findings of the framework analysis by computing descriptive statistics using R Statistical Software (version 4.3.3) [92] and RStudio (version 2023.12.1.402) [93]. Specifically, we calculated the proportion of activities in the HEALTH-CORP-FB typology (overall and within each domain of corporate influence) that had assigned ESG fields within each ESG framework. We also calculated means to determine the average number of activities with corresponding ESG fields (overall and within each domain) across all ESG frameworks.

For the ESG field classifications, we first calculated the inter-rater reliability of the relevance classifications using Gwet’s AC2 statistic, which we computed using the R package “irrCAC” version 1.0 [94]. Gwet’s AC2 was chosen to assess inter-rater agreement because it is robust to the prevalence of categories and can incorporate ordinal weights, such that agreements between adjacent ratings (e.g., medium vs. high) are penalized less than extreme disagreements (e.g., low vs. high) [95,96]. We interpreted the value of AC2 by benchmarking the result to the Landis-Koch scale [97] using the benchmarking method recommended by Gwet [98]. We did not calculate inter-rater reliability for type of business operations as the ratings were not assigned independently. Next, we computed the number and proportion of assigned ESG fields that corresponded to each category of the two attributes (relevance and type of business operations) as a function of the domain of corporate influence.

We produced Heatmaps in R to summarize and visualize the findings using the ggplot2 package version 3.5.1 [99], as was performed by O’Hearn and colleagues [57].

**Table 2:** Scoring attributes, definitions, and examples of ESG fields classified in the corresponding attribute category ^a^.

Scoring Attribute	Attribute Categories	Category Definitions	Example ESG Field
**Relevance** **^b^**	Low	Limited relevance and/or limited specificity	^c^ FB-PF-430a.1 [100]: Percentage of food ingredients sourced that are certified to third-party environmental or social standards, and percentages by standard [standards on child labor is provided as an example] [SASB Standards]
	Medium	Somewhat relevant and specific	^c^ Labor and Human Rights Controversies: The number of cases (severe controversies deemed major scandals or systematic risk incidents by RepRisk) occurring globally that pertain to human rights and/or labor rights violations in the company’s supply chain, as reported or discussed by influential news sources over the past three years. [JUST Capital Rankings]
	High	Highly relevant and specific	^c^ Disclosure 408-1 [101]: Operations and suppliers at significant risk for incidents of child labor. The reporting organization shall report the following information: Operations and suppliers considered to have significant risk for incidents of: (i) child labor; and (ii) young workers exposed to hazardous work. Operations and suppliers considered to have significant risk for incidents of child labor either in terms of: (i) type of operation (such as manufacturing plant) and supplier; and (ii) countries or geographic areas with operations and suppliers considered at risk. Measures taken by the organization in the reporting period intended to contribute to the effective abolition of child labor. [GRI Standards]
**Type of Business** **Operations ^d^**	Commitment	Public commitments (i.e., agreements, pledges, or promises) the company has made with respect to the issue in question.	Labor and Human Rights Commitment: whether the company discloses a human rights statement or policy on their website and the content of this statement [JUST Capital Rankings]
	Process	Processes, policies, strategies, evaluations, or targets the company has employed related to the respective topic. Metrics in this category may also measure structural features of the company, such as the extent of government ownership.	FB-PF-260a.2 [100]: Discussion of the process to identify and manage products and ingredients related to nutritional and health concerns among consumers. [SASB Standards]
	Performance	The result/outcome the company has obtained with respect to the issue in question.	FB-PF-140a.1 [100]: (1) Total water withdrawn, (2) total water consumed; percentage of each in regions with High or Extremely High Baseline Water Stress [SASB Standards]
	Controversies	Noteworthy events with potential negative impacts on stakeholders that are likely to damage the company’s public reputation.	Product Health and Environment Controversies: the number of cases of severe controversies occurring in the U.S. related to the health and environmental impacts of companies’ products and services. [JUST Capital Rankings]
	Non-Compliance and Penalties	The company’s non-compliance with regulatory frameworks and/or financial or non-financial penalties (fines, legal fees, settlements, sanctions, etc.) enforced upon the company due to their performance related to the respective topic.	Disclosure 416-2 [102] Incidents of non-compliance concerning the health and safety impacts of products and services. The reporting organization shall report the following information: a. Total number of incidents of non-compliance with regulations and/or voluntary codes concerning the health and safety impacts of products and services within the reporting period, by [various categories]. [GRI Standards]
	Disclosure	The company publicly reports information related to the respective topic.	Materiality Disclosure: Do you publicly disclose details of your materiality analysis, including information on how you conduct the materiality analysis process and your progress towards your targets or metrics? [S&P Global ESG Scores]

^a^ The scoring attributes applied in this study were adapted from those developed by O’Hearn and colleagues [57]. ^b^ Relevance refers to the applicability and specificity of the ESG field to the assigned HEALTH-CORP-FB activity. ^c^ These ESG fields were considered of low, medium, or high relevance to the following HEALTH-CORP-FB activity: Determine the use of child or forced labor directly or in the supply chain. ^d^ Type of Business Operations refers to the type of business activities the respective ESG field captures.

## 3. Results

### 3.1. Characteristics of Assigned ESG Fields

We assigned a total of 1348 ESG fields (i.e., disclosures or rating metrics) to activities in the HEALTH-CORP-FB typology. Among them, many metrics were repeated across subindustries or assigned to multiple activities while 499 (37%) were unique. The latter includes 13 unique metrics which were not mapped to a specific HEALTH-CORP-FB activity but were assigned to at least one of the overarching domains.

By domain, the highest number of ESG fields were mapped to Environmental Practices (419, 31%) whereas the lowest number were assigned to the Economic Practices domain (43, 3%). The S&P Global ESG Scores contributed the highest number of assigned fields (465/1348, 35%) whereas the JUST Capital Rankings supplied the lowest (123, 9%) (Table S5).

A total of 594 fields (387 unique) in the packaged foods subindustry that were assigned to the HEALTH-CORP-FB typology were further classified by relevance and type of business operations. Inter-rater agreement on the relevance classifications was “substantial” (AC2: 0.681 [95% CI: 0.624, 0.737]). Overall, 60% of classified metrics (357/594) were considered highly relevant to the assigned HEALTH-CORP-FB activity, 27% (162/594) were considered to have medium relevance, and 8% (46/594) were considered to have low relevance. A relevance classification was not applicable for 29 fields (5%) because the field was assigned to an overarching domain rather than a specific activity. The ESG fields most often captured processes (311/594, 53%), followed by performance (257, 43%), disclosure (147, 25%), non-compliance and penalties (56, 9%), controversies (49, 8%), and commitments (26, 4%). Percentages do not sum to 100 because ESG fields could be classified into more than one type of business operations.

### 3.2. Coverage of Activities in the HEALTH-CORP-FB Typology

**Figure 2:**
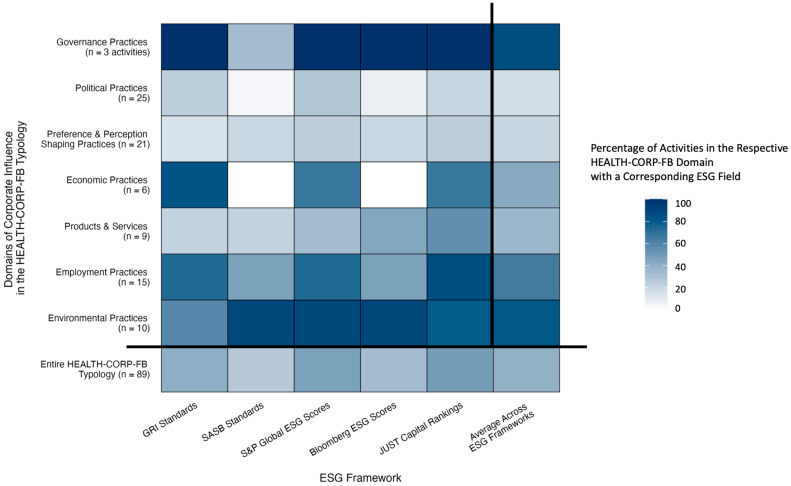
Heatmap of the percentage of activities in the HEALTH-CORP-FB typology with an assigned ESG field by domain of corporate influence and ESG framework.

On average, the five ESG frameworks had fields pertaining to less than half of the activities in the HEALTH-CORP-FB typology (34.8/89 activities, 39%; range across frameworks: 27–48%) (Table 3; Figure 2; Table S2). Only 14 activities (16%) had corresponding ESG fields from all five frameworks (e.g., water extraction) and 58 activities (65%) had a corresponding ESG field from at least one of the ESG frameworks (e.g., provision of medical benefits to employees).

Thirty-one activities in the HEALTH-CORP-FB typology did not have an assigned field from any of the five ESG frameworks. These activities included: “exploitation of revolving doors (i.e., employees who move between positions in industry and government)” (political practices), “taking or threatening legal action in response to unfavourable policies” (political practices), “the development or use of features of product packaging that are appealing to children (e.g., toys, colours)” (preference and perception-shaping practices), and “support for breastfeeding in the workplace” (employment practices) (Table S2). The lack of metrics dedicated to measuring these activities indicate key gaps in the extent to which the ESG frameworks in this study capture the activities of the F&B industry that impact population health.

**Table 3:** Number of activities in the HEALTH-CORP-FB typology with a corresponding ESG field by domain of corporate influence and ESG framework.

		Number of HEALTH-CORP-FB Activities (% of Total in Each Domain) with a Corresponding ESG Field:					
ESG Framework/ Domains of Corporate Influence	Total Number of Activities in Domain	GRI Standards	SASB Standards	S&P Global ESG Scores	Bloomberg ESG Scores	JUST Capital Rankings	Mean Number (%) of Activities with ESG Fields Across the ESG Frameworks
Governance Practices	3	3 (100)	1 (33)	3 (100)	3 (100)	3 (100)	2.6 (87)
Political Practices	25	6 (24)	1 (4)	7 (28)	2 (8)	5 (20)	4.2 (17)
Preference and Perception Shaping Practices	21	3 (14)	4 (19)	5 (24)	4 (19)	5 (24)	4.2 (20)
Economic Practices	6	5 (83)	0 (0)	4 (67)	0 (0)	4 (67)	2.6 (43)
Products and Services	9	2 (22)	2 (22)	3 (33)	4 (44)	5 (56)	3.2 (36)
Employment Practices	15	11 (73)	7 (47)	11 (73)	7 (47)	13 (87)	9.8 (65)
Environmental Practices	10	6 (60)	9 (90)	9 (90)	9 (90)	8 (80)	8.2 (82)
Entire HEALTH-CORP-FB Typology	89	36 (40)	24 (27)	42 (47)	29 (33)	43 (48)	34.8 (39)

By domain, the ESG frameworks covered a larger proportion of the activities in the Governance, Environmental, Employment, and Economic Practices domains (range across domains: 43–87%) than the Products and Services, Preference and Perception-Shaping Practices, and Political Practices domains (17–36%) (Table 3; Figure 2; Table S2).

**Figure 3:**
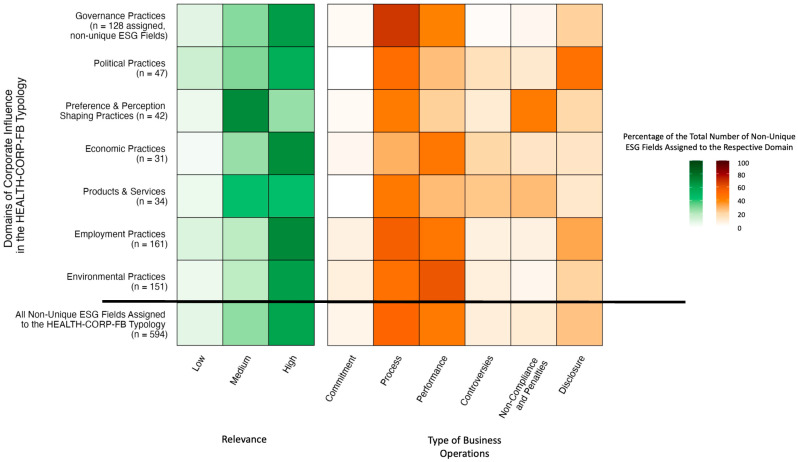
Heatmaps of the characteristics of the assigned ESG fields (Packaged Foods subindustry) as a function of the domain of corporate influence.

Moreover, higher proportions of ESG fields assigned to activities in the former domains were considered highly relevant (range across domains: 21–112 fields, 62–70%) compared to those assigned to the latter domains (range: 11–25 fields, 26–53%) (Table 4, Figure 3). ESG fields assigned to the former domains were also more likely to measure corporate performance (14–89 fields, 40–59%) than the fields assigned to the latter domains (9–12 fields, 21–26%) (Table 4, Figure 3). Instead of focusing on corporate performance, ESG fields mapped to the Political Practices domain commonly measured processes (e.g., provision of anti-corruption training) and disclosure (e.g., disclosure of political donations) and fields mapped to the Preference and Perception-Shaping Practices domain commonly measured processes (e.g., responsible advertising policy in place) and non-compliance and penalties (e.g., incidents of non-compliance with marketing codes). In addition to fields measuring processes (15/34, 44%) (e.g., reformulation), the Products and Services domain had a similar number of fields that monitored performance (9, 26%), controversies (8, 24%), and non-compliance and penalties (9, 26%).

Additional findings, such as the characteristics of the ESG fields as a function of the ESG framework from which they were extracted, are reported in the Supplementary Material for brevity (Tables S5–S11).

**Table 4:** Characteristics of the assigned ESG Fields (packaged food subindustry) as a function of the domain of corporate influence.

Number of Non-Unique ESG Fields Corresponding to Attribute Category and Domain (% of Total Non-Unique Fields Assigned to Domain) ^a^:											
		Relevance			Type of Business Operations ^b^						
Domain of Corporate Influence	Total Number of Non-Unique Fields Assigned to Domain	Low	Medium	High	N/A ^c^	Commitment	Process	Performance	Controversies	Non-Compliance and Penalties	Disclosure
Governance Practices	128	11 (9)	37 (29)	80 (63)	0 (0)	3 (2)	87 (68)	51 (40)	2 (2)	4 (3)	27 (21)
Political Practices	47	7 (15)	14 (30)	25 (53)	1 (2)	0 (0)	23 (49)	12 (26)	7 (15)	5 (11)	22 (47) ^d^
Preference and Perception Shaping Practices	42	2 (5)	29 (69)	11 (26)	0 (0)	1 (2)	18 (43)	9 (21)	4 (10)	18 (43)	8 (19)
Economic Practices	31	1 (3)	8 (26)	21 (68)	1 (3)	1 (3)	9 (29)	14 (45)	6 (19)	4 (13)	4 (13)
Products and Services	34	2 (6)	15 (44)	15 (44)	2 (6)	0 (0)	15 (44)	9 (26)	8 (24)	9 (26)	4 (12)
Employment Practices	161	16 (10)	31 (19)	112 (70)	2 (1)	10 (6)	88 (55)	73 (45)	11 (7)	10 (6)	51 (32)
Environmental Practices	151	7 (5)	28 (19)	93 (62)	23 (15)	11 (7)	71 (47)	89 (59)	11 (7)	6 (4)	31 (21)

^a^ Duplicative ESG fields (i.e., those assigned to more than one HEALTH-CORP-FB activity) are counted separately in this table. This is because the relevance classification is dependent on the assigned activity. However, similar distributions across domains are observed for the type of business operations when only the unique ESG fields are counted within each domain (Table S10). ^b^ The counts for type of business operations do not sum to the total metric count because metrics could be classified to more than one type of business operations. For the same reason, the corresponding percentages do not sum to 100. ^c^ Some metrics did not receive a relevance classification because they were not assigned to a specific activity; rather, they were assigned to the domain itself. ^d^ 21 of the 47 non-unique metrics assigned to the Political Practices domain (47%) came from the S&P Global ESG Scores. 60% of the assigned fields from S&P Global measure disclosure (in addition to other types of business operations) (Table S8), which may account in part for the significant proportion of metrics in this category that measure disclosure. Moreover, one of the HEALTH-CORP-FB activities in the Political Practices domain is focused specifically on disclosure (i.e., place restrictions on access to corporate data).

## 4. Discussion

In this study, we examined five ESG frameworks to determine the extent to which they capture the activities of the F&B industry that influence population health and health equity across several dimensions (e.g., nutritional, environmental, employment, political). The ESG frameworks included in this study capture some of the activities of the F&B industry that affect population health and health equity (e.g., water use and extraction, waste production and management, occupational injuries, non-compliance with marketing practices), especially with respect to Governance, Environmental, Employment, and Economic Practices. However, critical gaps were identified in both the coverage and measurement of F&B companies’ Products and Services, Preference and Perception-Shaping Practices, and Political Practices.

Specifically, there is insufficient focus on the characteristics of F&B company products, as evidenced by the lack of metrics dedicated to food processing, portion size, price, and the physical availability of F&B products (Table S2). In their study of ESG nutrition metrics, O’Hearn and colleagues similarly identified that metrics focused on product healthfulness were limited in scope (e.g., focused on single nutrients) and that there were a dearth of metrics dedicated to assessing product distribution, a key driver of nutrition-related health inequities [57]. Given the significant contribution of the F&B industry to the non-communicable disease (NCD) crisis [103,104,105,106], these deficits are critical. If ESG frameworks aim to sufficiently measure the social performance of this industry, accurately and comprehensively assessing the health impacts of F&B products is a necessity.

Second, the ESG frameworks in this study did not often address several established mechanisms that F&B companies use to undermine public health efforts, such as lobbying, exploitation of revolving doors (i.e., employees who move between the public and private sector [107]), and the use of litigation to delay or prevent the implementation of public health policies (Table S2) [1,4,71,108,109]. The aggressive political activity of the F&B industry has been identified as a critical barrier to the implementation of evidence-based regulations designed to reduce the burden of NCDs [1,4,71,108,109]. Failing to measure these activities constitutes a major blind spot in determining the “social” impact of F&B companies.

Finally, when activities in the Products and Services, Political Practices, and Preference and Perception-Shaping domains were measured, they appeared to be measured in an inferior way. Rather than focusing on actual corporate performance (e.g., amount spent on lobbying), the ESG metrics focused on these activities often captured corporate processes (e.g., company provides anti-corruption training to employees), disclosure (e.g., company discloses its political donations), or non-compliance with regulatory frameworks (e.g., fines received for marketing breaches). While these types of indicators may shed light on past or future corporate behavior, they do not provide direct information about the company’s current performance in these areas.

These findings generally correspond with previous research on this topic. O’Hearn and colleagues [57] similarly identified gaps in the assessment of F&B company products, such as their accessibility and affordability; these gaps were also supported by an evaluation of the ISS ESG rating system conducted by Robinson and colleagues [110]. Our findings are similarly congruent with an analysis conducted by the GRI and the Robert Wood Johnson Foundation (RWJF) [66], in which they assessed the extent to which seven ESG frameworks capture 15 Culture of Health Business Practices (COHBPs) (e.g., health insurance). The authors identified that occupational health and safety issues and environmental impacts were covered in the most detail whereas topics such as responsible corporate political activity were not as commonly captured. Finally, a series of Fixing the Business of Food (FTBF) reports published by the Columbia Center on Sustainable Investment (and partners) assessed the congruence of ESG frameworks and non-financial reporting by the F&B industry with the Sustainable Development Goals (SDGs) [63,64,65]. In agreement with the findings from this study, they concluded that there was a lack of attention paid to tax, litigation, and lobbying activities within ESG frameworks and/or sustainability reports issued by F&B companies.

### 4.1. Recommendations for ESG Framework Reform

Importantly, our goal is not for the HEALTH-CORP-FB typology to replace existing ESG frameworks as it is focused on the health impacts of F&B companies specifically and was not built for that purpose. Rather, evaluating the ESG frameworks against the HEALTH-CORP-FB typology allowed us to identify opportunities for high-quality, evidence-based metrics to be added to existing ESG frameworks to improve the extent to which they capture the health impacts of the F&B industry.

First, we recommend that ESG frameworks assess the extent to which a commercial entity relies on ultra-processed foods for revenue generation. Ultra-processed foods have been defined as “industrially manufactured food products made up of several ingredients (formulations) including sugar, oils, fats and salt (generally in combination and in higher amounts than in processed foods) and food substances of no or rare culinary use (such as high-fructose corn syrup, hydrogenated oils, modified starches and protein isolates).” [111] A recent Lancet series concluded that ultra-processed foods have disrupted traditional diets and culinary practices and that their consumption leads to a significant worsening of dietary quality through mechanisms such as nutrient imbalances, reduced intake of health-protective phytochemicals, and increased intake of toxic compounds [112]. The series also concluded that this degradation of dietary quality through ultra-processed food consumption leads to increased risk of several chronic diseases, such as cardiovascular disease, type 2 diabetes, and depression [112]. Ultra-processed foods may also have significant environmental impacts through extensive packaging and energy demand during the processing phase [113,114].

To calculate revenue from ultra-processed foods, companies could apply the Nova classification, which groups F&B products into four main categories of processing (unprocessed, processed culinary ingredients, processed foods, and ultra-processed foods) [111], to their product lines. This could be carried out using the official Nova application guidance or by leveraging the Nova classifications of branded products already provided by the Open Food Facts database [111,115]. To reduce the risk that companies apply the Nova classification in a way that leads to biased results, trained public health professionals could be tasked with verifying the resulting classifications. Reporting separately on revenue generation from ultra-processed foods would allow for a clearer picture of product improvement in relation to processing when compared to reporting an aggregated Nutrient Profiling Score (NPS) only, as has been previously suggested by others [57,58,116].

We also strongly recommend that ESG reporting frameworks require F&B companies to disclose information in their ESG reports about their annual financial or in-kind contributions for lobbying activities, litigation the company has engaged in pertaining to proposed public health measures, and the number and percentage of senior employees who have worked in government agencies in the last ten years (i.e., “revolving doors”). Lobbying, litigation, and revolving doors are consistently documented as key strategies that F&B companies use to block or influence evidence-based public health policies (e.g., front-of-pack labeling) [71,117,118]. They are also some of the most difficult activities to track, as available data sources are often patchy, time consuming to analyze, and do not provide sufficient detail [119,120]. While litigation activities may be described in some company documents (e.g., financial reports) [121,122], disclosing these activities within ESG reports allows the societal impacts of these activities to be considered, not just their potential financial implications. In general, embedding performance-based indicators of litigation and lobbying activities and revolving doors within ESG reporting frameworks and rating systems could meaningfully improve transparency and enhance corporate accountability for political engagement. Table S12 provides other recommended metrics and their corresponding public health rationale.

Companies often report that ESG data collection and reporting is overly burdensome [46] and investors refer to “issue overload” [123] as a barrier to more robust consideration of health issues within ESG investment analysis. For this reason, we are not necessarily proposing a net increase in ESG metrics. Rather, health-focused indicators could replace less relevant existing indicators. For example, high-quality information about the characteristics of F&B products (e.g., nutritional composition, extent of processing) is arguably much more relevant to the F&B industry’s social impact (and associated investment risk and opportunity) than their consumer data protection policies (an issue commonly measured by the ESG frameworks investigated in this study).

However, replacing indicators is only part of the challenge. Additional research is needed to understand barriers to integrating health-related fields into ESG frameworks. Evidence from Chan and colleagues [116] illustrates this complexity: while nutrition-related reporting metrics were welcomed by Australian food companies, respondents identified significant obstacles, including limited company buy-in, resource constraints, and concerns about public perception. The authors concluded that mandatory reporting regulations may be necessary to achieve consistent and adequate nutrition-related disclosures across companies [116].

### 4.2. Implications for Key Stakeholders

The findings of this study have important implications for ESG framework producers, F&B companies, policy makers, and the public health community. ESG data is facing a crisis of credibility because it has failed to adequately distinguish between companies that are performing well on ESG issues and those that are not [26,27,46,124]. S&P Global, for example, faced public criticism after it was revealed that its ESG rating algorithm allowed tobacco companies such as Philip Morris International to receive high ESG scores, despite the significant health impact of the tobacco industry [125]. Moreover, there is growing recognition of the extensive interconnectedness between human health and the health of Earth’s natural systems, an idea encapsulated by the popular concept of planetary health [126]. These connections are especially prominent when considering the impact of human diets on both human health and the physical environment [127]. Working to advance sustainable food systems through ESG screening without considering the health impacts of these decisions is like trying to run while only wearing one shoe—you will not get very far. Therefore, integrating high-quality ESG indicators that capture corporate health impacts into existing frameworks could significantly enhance the quality of ESG products and increase their value to both investors and other key stakeholders.

F&B companies also stand to gain from the development of rigorous disclosures of health impact. Companies that are already managing their health impact effectively can be rewarded for it. Moreover, F&B companies that seek to gain an advantage within a competitive global food industry have another avenue to differentiate themselves from the pack. The race towards becoming a “health-promoting” company can lead to a plethora of opportunities for companies in the same way that the sustainability movement has opened up new avenues to earn consumer trust and brand loyalty [128]. As proposed by Chan and colleagues [116], however, mandatory reporting regulations are likely required to ensure that F&B companies report on the health impacts in a consistent, accurate, and transparent manner.

Finally, higher-quality ESG disclosures of corporate health impact offer the public health community a much-needed mechanism to monitor and address the CDOH. A key barrier existing corporate monitoring efforts face is the lack of publicly accessible data [10,129,130,131]. The findings from this study indicate some topics for which existing ESG reports may already contain relevant data points (e.g., occupational injuries and fatalities, water extraction, payment of living wages) (Table S2). Though these data points are not always reported in a way that is easy to aggregate, digital reporting taxonomies (e.g., the GRI’s sustainability taxonomy) are being developed that will allow ESG data to be reported in machine-readable formats with corresponding digital tags that could facilitate automated data extraction [132].

Such monitoring data could empower stakeholders to hold F&B companies accountable for their health impacts [133]. Public health organizations could use health-focused ESG disclosures to identify areas requiring stronger regulation to protect population health. Civil society organizations might leverage this information to drive advocacy campaigns and consumer boycotts that pressure companies to improve practices [19]. Similarly, shareholder advocacy organizations like ShareAction [134], the Access to Nutrition Initiative (ATNI) [135] and the Interfaith Center on Corporate Responsibility [136] can use these disclosures to strengthen shareholder engagement. For instance, ATNI’s “Investor Expectations on Nutrition, Diets, & Health” [135] initiative encourages institutional investors to engage F&B companies on issues such as nutrition strategy and governance, responsible lobbying, and improved transparency for stakeholders. Access to more detailed data on the health-related activities of individual F&B companies would enable these investors to engage in a more targeted and effective manner.

Including health-focused ESG indicators within existing rating systems could also create a powerful financial incentive for F&B companies to improve their practices. Poor ESG ratings can negatively affect a company’s cost of capital by signaling higher risk to investors and lenders compared to firms with strong ESG performance [62,137,138]. As a result, shareholders may demand higher returns, and lenders may impose higher interest rates to compensate for perceived risk [62,137,138]. This increases the overall cost of doing business for companies with weak ESG performance, creating a potential behavioral incentive to improve [62,137,138]. Evidence suggests that some firms change their practices in response to ESG assessments, for example, by improving their waste management practices [59,60,139,140,141,142,143,144]. As ESG investment analysis matures and disclosure practices become more robust, this incentive is likely to strengthen [59], offering a valuable opportunity to drive corporate behavior change.

In addition to these opportunities, the increasing popularity of ESG investment analysis creates an obligation for the public health community and other key stakeholders to ensure that ESG health disclosures do not inadvertently reward health-harming companies (e.g., tobacco companies), critical health impacts are recognized, and ESG disclosures do not become yet another tool for companies to suggest that they are able to effectively self-regulate [49,145,146,147].

### 4.3. Strengths and Limitations of This Study

The advantages of this study include that we used systematic methods to identify the strengths and weaknesses of five existing ESG frameworks (standards and ratings providers) concerning how they capture the activities of the F&B industry that affect population health. In applying a novel lens to our investigation (i.e., the HEALTH-CORP-FB typology), we were able to add to existing work by documenting previously unidentified gaps, such as the lack of ESG metrics dedicated to measuring the number of employees that move between the public and private sector (i.e., revolving doors). We also provide actionable recommendations to improve the quality of ESG disclosures as they pertain to health. These recommendations may directly inform efforts to update ESG standards, such as the GRI’s plans to develop an industry-specific ESG standard for the F&B industry [148,149].

Our study also has several limitations. First, our sample does not contain the entire universe of ESG reporting standards and ratings providers, and our findings may not be generalizable to other ESG frameworks. Prominent ESG rating systems produced by MSCI and Sustainalytics were not included because our research team was not granted access or were quoted a significant fee to access these frameworks. S&P Global’s Beverages Industry was not included in our analysis because the associated CSA document was not publicly available. Moreover, newer versions of the CSA (i.e., post-2021) may contain additional fields relevant to the HEALTH-CORP-FB typology (e.g., employee support programs) [150]. ESG frameworks are rapidly evolving, and therefore, the findings from this study represent the state of ESG frameworks at a specific point in time (i.e., those that could be collected from September 2023 to March 2024). The industry-specific ESG frameworks we investigated use different definitions of the F&B industry and associated subindustries, and therefore, they may not be directly comparable. The SASB and GRI Standards allow companies to exercise agency in what they report. Thus, the existence of a field does not guarantee disclosure by F&B companies.

Moreover, the usefulness of deductive framework analysis depends on the quality and completeness of its underlying framework. Though the HEALTH-CORP-FB typology was developed using a rigorous research process, it likely does not identify every single activity through which the F&B industry can influence population health and health equity. For example, food safety was not included in the HEALTH-CORP-FB typology, though it is relevant to human health and was included in several ESG frameworks. As the HEALTH-CORP-FB typology is relatively new, it is still evolving and may require iterative refinement. Importantly, some of the activities in the HEALTH-CORP-FB typology (e.g., employing argumentative strategies within policy submissions) may also not be amenable to self-report or measurement using ESG metrics. Finally, we did not assess the validity of the ESG fields or the ways the fields were weighted and aggregated to form overarching ESG scores; these are important topics for future inquiry.

## 5. Conclusions

In this study, we evaluated the extent to which five existing ESG frameworks capture the activities of the F&B industry that can influence population health and health equity. The ESG frameworks investigated in this study capture some F&B industry activities that are relevant to population health, particularly with respect to their Governance, Environmental, Employment, and Economic practices (e.g., water use and extraction, waste production and management, occupational injuries). However, the findings also indicate the presence of key gaps concerning the coverage and measurement of some F&B industry activities that are relevant to population health, particularly regarding their Products and Services, Preference and Perception-Shaping Practices, and Political Practices (e.g., the lack of metrics dedicated to food processing, litigation practices, and lobbying practices). Specific metrics can be added to existing ESG frameworks to improve the extent to which they capture the health impacts of the F&B industry (e.g., extent of revenue generation from ultra-processed foods). Stronger measurement of F&B industry health impacts within ESG investment frameworks may provide a useful mechanism to increase the accountability of the F&B industry and encourage F&B companies to improve their health impacts.

## Data Availability

The ESG fields assigned to the HEALTH-CORP-FB typology and the classifications of each of these fields by relevance and type of business operations can be found on Zenodo at https://doi.org/10.5281/zenodo.15022024. Data from some of the ESG frameworks is not provided as this data is not publicly available.

## Supplementary Materials

The following supporting information can be downloaded at: https://www.mdpi.com/article/10.3390/ijerph23010030/s1, Table S1: List of Documents Used to Extract ESG fields and Associated Version Dates; Table S2: The findings of the framework analysis at the level of individual activities in the HEALTH-CORP-FB typology; Table S3: Definitions of the Domains of Corporate Influence in the HEALTH-CORP-FB Typology; Table S4: Definitions of the SASB ‘Processed Foods’ subindustry, Bloomberg ‘Packaged Food’ subindustry, and the S&P Global ‘Food Products’ subindustry; Table S5: Additional Text Results (Distribution of Assigned Fields Across Domains and ESG Frameworks); Table S6: Additional Text Results (Coverage of HEALTH-CORP-FB Activities by ESG Framework); Table S7: Heatmaps of the characteristics of the assigned ESG fields (Packaged Foods subindustry) as a function of the ESG framework; Table S8: Characteristics of the assigned ESG Fields (Packaged Foods subindustry) as a function of the ESG framework; Table S9: Additional Text Results (Characteristics of the ESG Fields as a Function of the ESG Framework); Table S10: Distribution of the Type of Business Operations and Data Type Attributes for Assigned ESG Fields (Packaged Food Subindustry) as a Function of the Domain of Corporate Influence [*Unique ESG Fields Only*]; Table S11: Distribution of the Type of Business Operations and Data Type Attributes for Assigned ESG Fields (Packaged Food Subindustry) as a Function of the ESG Framework [*Unique Metrics Only*]; Table S12: Recommendations for disclosures/metrics that could be used to address gaps in existing ESG frameworks included in this study. References [151,152,153,154,155,156,157,158,159,160,161,162,163,164,165,166,167,168,169,170,171,172,173,174,175,176,177,178,179,180] are cited in the Supplementary Materials.

## Abbreviations

CDOH	Commercial determinants of health
ESG	Environmental, Social, and Governance
ESRS	European Sustainability Reporting Standards
F&B	Food and beverage
GRI	Global Reporting Initiative
HEALTH-CORP-FB	Corporate Influences on Population Health (HEALTH-CORP)—Food and Beverage
IFRS	International Financial Reporting Standards
ISSB	International Sustainability Standards Board
SASB	Sustainability Accounting Standards Board
SDGs	Sustainable Development Goals
NPS	Nutrient Profiling Score
