# Supplementary material for: Do ESG Frameworks Capture Corporate Health Impacts? An Analysis of the Food and Beverage Industry
Source: Int J Environ Res Public Health. 2025 Dec 24;23(1):30. doi: 10.3390/ijerph23010030 (PMC12840626; doi:10.3390/ijerph23010030)
Supplement: Supplementary file 1 [file ijerph-23-00030-s001.zip › ijerph-3975226-supplementary.pdf]

Supporting Information

Table of Contents

Table S1. List of Documents Used to Extract ESG fields and Associated Version Dates............1

Table S2. The findings of the framework analysis at the level of individual activities in the HEALTH-CORP-FB typology.[1] 4

Table S3. Definitions of the Domains of Corporate Influence in the HEALTH-CORP-FB Typology.[1] 11

Table S4. Definitions of the SASB 'Processed Foods' subindustry, Bloomberg 'Packaged Food' subindustry, and the S&P Global 'Food Products' subindustry. 12

Table S5. Additional Text Results (Distribution of Assigned Fields Across Domains and ESG Frameworks). 12

Table S6. Additional Text Results (Coverage of HEALTH-CORP-FB Activities by ESG Framework). 13

Table S7. Heatmaps of the characteristics of the assigned ESG fields (Packaged Foods subindustry) as a function of the ESG framework. 14

Table S8. Characteristics of the assigned ESG Fields (Packaged Foods subindustry) as a function of the ESG framework. 15

Table S9. Additional Text Results (Characteristics of the ESG Fields as a Function of the ESG Framework). 16

Table S10. Distribution of the Type of Business Operations and Data Type Attributes for Assigned ESG Fields (Packaged Food Subindustry) as a Function of the Domain of Corporate Influence [*Unique ESG Fields Only*] 17

Table S11. Distribution of the Type of Business Operations and Data Type Attributes for Assigned ESG Fields (Packaged Food Subindustry) as a Function of the ESG Framework [*Unique Metrics Only*] 18

Table S12. Recommendations for disclosures/metrics that could be used to address gaps in existing ESG frameworks included in this study.[a] 19

Table S1. List of Documents Used to Extract ESG fields and Associated Version Dates

ESG Framework	Documents Used to Extract ESG Fields	Version Date of Documents Used in This Study
Global Reporting Initiative (GRI)	GRI 1: Foundation 2021	January 1, 2023
	GRI 2: General Disclosures 2021	January 1, 2023
	GRI 3: Material Topics 2021	January 1, 2023
	GRI 201: Economic Performance	July 1, 2018
	GRI 202: Market Presence	July 1, 2018
	GRI 203: Indirect Economic Impacts	July 1, 2018
	GRI 204: Procurement Practices	July 1, 2018
	GRI 205: Anti-corruption	July 1, 2018
	GRI 206: Anti-competitive Behavior	July 1, 2018
	GRI 207: Tax	January 1, 2021
	GRI 301: Materials	July 1, 2018
	GRI 302: Energy	July 1, 2018
	GRI 303: Water and Effluents	January 1, 2021
	GRI 304: Biodiversity	July 1, 2018
	GRI 305: Emission 2016	July 1, 2018
	GRI 306: Effluents and Waste 2020	July 1, 2018
	GRI 306: Waste 2020	January 1, 2022
	GRI 308: Supplier Environmental Assessment 2016	July 1, 2018
	GRI 401: Employment 2016	July 1, 2018
	GRI 402: Labor – Management Relations 2016	July 1, 2018
	GRI 403: Occupational Health and Safety 2018	January 1, 2021
	GRI 404: Training and Education 2016	July 1, 2018

	GRI 405: Diversity and Equal Opportunity 2016	July 1, 2018
	GRI 406: Non-discrimination 2016	July 1, 2018
	GRI 407: Freedom of Association and Collective Bargaining 2016	July 1, 2018
	GRI 408: Child Labor 2016	July 1, 2018
	GRI 409: Forced or Compulsory Labor 2016	July 1, 2018
	GRI 410: Security Practices 2016	July 1, 2018
	GRI 411: Rights of Indigenous Peoples 2016	July 1, 2018
	GRI 413: Local Communities 2016	July 1, 2018
	GRI 414: Supplier Social Assessment 2016	July 1, 2018
	GRI 415: Public Policy 2016	July 1, 2018
	GRI 416: Customer Health and Safety 2016	July 1, 2018
	GRI 417: Marketing and Labeling 2016	July 1, 2018
	GRI 418: Customer Privacy 2016	July 1, 2018
Sustainability Accounting Standards Board (SASB)	Agricultural Products Sustainability Accounting Standard	June 2023
	Food Retailers & Distributors Sustainability Accounting Standard	June 2023
	Meat, Poultry & Dairy Sustainability Accounting Standard	June 2023
	Non-Alcoholic Beverages Sustainability Accounting Standard	June 2023
	Processed Foods Sustainability Accounting Standard	June 2023
	Restaurants Sustainability Accounting Standard	June 2023
S&P Global ESG Scores	S&P Global Corporate Sustainability Assessment: Food Products Questionnaire and Rationale	2021
	S&P Global Corporate Sustainability Assessment: Restaurants & Leisure Facilities Questionnaire and Rationale	2021
	S&P Global Corporate Sustainability Assessment: Food & Staples Retailing Questionnaire and Rationale	2021
Bloomberg ESG Scores[a]	Environmental and Social Scores Methodology Industry Guide - Packaged Food & Beverages	November 2021
	Environmental and Social Scores Methodology Industry Guide – Retail and Wholesale	November 2021
	Environmental and Social Scores Methodology Industry Guide – Agriculture	June 2021

	Environmental and Social Scores Methodology Industry Guide – Restaurants	August 2022
	Governance Scores Methodology Theme Guide – Shareholder Rights	February 2024
	Governance Scores Methodology Theme Guide – Executive Compensation	February 2024
	Governance Scores Methodology Theme Guide – Board Composition	February 2024
	Governance Scores Methodology Theme Guide – Audit	February 2024
JUST Capital Rankings	JUST Capital Ranking Methodology	January 2023

[a]Definitions for each of the ESG fields in the Bloomberg methodology documents were obtained from an Excel spreadsheet available on the Bloomberg terminal (dated Jan 31st, 2024).

Table S2. The findings of the framework analysis at the level of individual activities in the HEALTH-CORP-FB typology [1].

	Global Reporting Initiative (GRI) Standards	Sustainability Accounting Standards Board (SASB) Standards	S&P Global ESG Scores	Bloomberg ESG Scores	JUST Capital Rankings	ESG Field Assigned From At Least One ESG Framework
**DISTAL DOMAINS OF CORPORATE INFLUENCE**						
**Governance Practices**						
Develop and maintain ownership and remuneration structures that determine how power and resources are distributed	Y		Y	Y	Y	Y
Pursue mergers, acquisitions, and joint ventures that consolidate the food industry	Y		Y	Y	Y	Y
Develop overarching strategies and conduct evaluations of operations to understand and improve health impact	Y	Y	Y	Y	Y	Y
**Political Practices**						
Activities related to securing a favourable policy environment:						
Engage in political financing	Y		Y		Y	Y
Engage in bribery	Y		Y		Y	Y
Advocate for policies to limit corporate liability for health harms						
Build relationships with public health institutions and/or other relevant groups	Y		Y			Y
Advocate for placement of corporate representatives on relevant associations and boards	Y					Y
Exploit the use of revolving doors (i.e., employees who move between positions in industry and government)						
Contribute to legal instruments via third parties, front groups, or coalitions						
Use corporate social responsibility (CSR) to gain access or influence policy making						

	GRI Standards	SASB Standards	S&P Global ESG Scores	Bloomberg ESG Scores	JUST Capital Rankings	ESG Field Assigned From At Least One ESG Framework
Exploit the weaker regulatory context and lower resource availability of low- and middle-income countries (LMICs)						
Take or threaten legal action in response to unfavourable policies						
Advocate for self-regulation, co-regulation, or voluntary codes						
Engage in lobbying, including via third parties			Y		Y	Y
Engage in product reformulation to avoid legislation or suggest the company is taking action independently		Y	Y	Y		Y
Create or exploit policy loopholes, jurisdictional overlaps, or technicalities to avoid unfavourable policies						
Leverage pre-emption tactics within national/regional governments						
Leverage trade and investment treaties to challenge unfavourable policies						
Amplify influence via front groups or membership in industry alliances/coalitions	Y		Y			Y
Employ argumentative strategies within policy submissions (e.g., questioning institutional mandates) to oppose and/or delay the implementation of unfavourable policies						
Misrepresent evidence or demand unrealistic standards of evidence within policy submissions						
Exploit or create divisions in the public health community						
Intimidate and/or discredit opponents						
Threaten to or shift operations to regions with weaker labour regulations						
Other political activities:						
Advocate for privatization of food service provision programs (e.g., school lunch programs)						
Place restrictions on access to corporate data			Y	Y	Y	Y
Expropriate land for industry activities (i.e., land grabs)	Y				Y	Y

	GRI Standards	SASB Standards	S&P Global ESG Scores	Bloomberg ESG Scores	JUST Capital Rankings	ESG Field Assigned From At Least One ESG Framework
**Preference & Perception Shaping Practices**						
Activities related to promoting products:						
Employ aggressive physical and digital marketing techniques to encourage product recognition and consumption	Y	Y		Y	Y	Y
Engage in marketing of harmful products in ways that disproportionately target disadvantaged or vulnerable groups		Y		Y	Y	Y
Develop and/or use features of product packaging (e.g., toys, colours) that are appealing to children						
Use incentive programs or exclusivity agreements to promote products within schools			Y		Y	Y
Sponsor sports, music, or other cultural events, individuals, and infrastructure						
Activities related to shaping the public debate about products & their health implications:						
Fund or deliver health education initiatives directed at the public			Y		Y	Y
Conduct educational and/or advocacy campaigns to influence the public's perception of health policies						
Craft and propagate inaccurate or skewed narratives about health & disease						
Advance the ideas of individual responsibility and consumer choice						
Advance misleading health claims	Y	Y		Y	Y	Y
Acquire ownership, establish relationships, or exert influence on the media via advertising spending						
Use experts and key opinion leaders to further industry interests in the public forum (e.g., social media)						

	GRI Standards	SASB Standards	S&P Global ESG Scores	Bloomberg ESG Scores	JUST Capital Rankings	ESG Field Assigned From At Least One ESG Framework
Activities related to shaping the professional debate about human health and nutrition:						
Fund professional associations	Y					Y
Contribute to the development of clinical standards						
Fund or deliver health education initiatives directed at health care providers or health professions students						
Activities related to shaping the production and interpretation of evidence:						
Conduct internal research and generate evidence about the health impacts of the company's strategies and products		Y	Y	Y		Y
Fund external stakeholders (e.g., scientists, think tanks) to conduct and/or disseminate research			Y			Y
Suppress, amplify, or cherry-pick evidence or attempt to generate uncertainty about unfavourable evidence						
Obscure conflicts of interest in research						
Contribute to the development of scientific standards						
Falsify or misrepresent data						
**Economic Practices**						
Engage in fair or unfair tax practices	Y		Y		Y	Y
Engage in profiteering or price fixing	Y		Y		Y	Y
Earn revenue and contribute to economic growth	Y				Y	Y
Use foreign-direct investment (FDI) practices to enter the markets of low- and middle-income countries			Y			Y
Replace local retailers and other local economic activities	Y		Y		Y	Y
Commercialize Indigenous crops and agricultural practices	Y					Y

	GRI Standards	SASB Standards	S&P Global ESG Scores	Bloomberg ESG Scores	JUST Capital Rankings	ESG Field Assigned From At Least One ESG Framework
**PROXIMAL DOMAINS OF CORPORATE INFLUENCE**						
**Products & Services**						
Activities related to the characteristics of products:						
Determine the nutritional properties of products	Y	Y	Y	Y	Y	Y
Determine the level of processing of products					Y	
Develop or promote products with addictive properties						
Determine the portion size of products				Y		Y
Determine the features of products that dictate its shelf-life						
Determine the comprehensiveness and accuracy of nutritional information on products	Y	Y	Y	Y	Y	Y
Activities related to the accessibility of products:						
Determine the price of healthy/unhealthy products and the use of price promotions, discounts, and/or coupons					Y	Y
Determine the physical proximity of products to consumers					Y	Y
Other product-related activities:						
Develop nutrition-related innovations and engage in associated patenting practices			Y	Y		Y
**Employment Practices**						
Activities related to the characteristics of employment:						
Determine the number of employment opportunities	Y	Y	Y		Y	Y
Determine the adequacy of pay in relation to local living standards	Y	Y	Y	Y	Y	Y
Determine the stability of employment and workers’ responsibility for risk	Y	Y	Y	Y	Y	Y
Engage in efforts to promote diversity, equity, and inclusion	Y		Y	Y	Y	Y

	GRI Standards	SASB Standards	S&P Global ESG Scores	Bloomberg ESG Scores	JUST Capital Rankings	ESG Field Assigned From At Least One ESG Framework
Activities related to the benefits received through employment:						
Determine the presence and quality of medical benefits	Y				Y	Y
Determine the presence and quality of pension plans	Y				Y	Y
Determine the presence, length, and paid or unpaid status of parental leave	Y		Y		Y	Y
Determine employee access to vacation leave					Y	Y
Provide access to childcare			Y		Y	Y
Activities related to the conditions of employment:						
Determine workers' exposure to physical hazards (e.g., pesticides, machinery) and associated safety protocols	Y	Y	Y	Y	Y	Y
Determine worker's exposure to psychosocial hazards (i.e., discrimination, social isolation, hostile work environments, long working hours, work-family imbalance) and associated supports	Y	Y	Y	Y	Y	Y
Determine workers' freedom to unionize or engage in collective bargaining without fear of reprisal	Y	Y	Y	Y	Y	Y
Determine the extent of support for breastfeeding in the workplace						
Develop opportunities to work remotely and determine the characteristics of remote work			Y			Y
Determine the use of child or forced labour directly or in the supply chain	Y	Y	Y	Y	Y	Y
**Environmental Practices**						
Extract and use water, including efforts to conserve water	Y	Y	Y	Y	Y	Y
Use (or avoid the use of) agrochemicals	Y	Y	Y	Y	Y	Y
Engage in harvesting that is within or outside of ecological bounds		Y	Y	Y	Y	Y
Contribute to land use and land use change, including efforts to conserve land	Y	Y	Y		Y	Y

	GRI Standards	SASB Standards	S&P Global ESG Scores	Bloomberg ESG Scores	JUST Capital Rankings	ESG Field Assigned From At Least One ESG Framework
Contribute to soil erosion and degradation, including efforts to prevent erosion and degradation			Y	Y		Y
Consume energy and determine the source, including efforts to conserve energy or use renewable sources	Y	Y	Y	Y	Y	Y
Produce and manage waste, including efforts to reduce waste	Y	Y	Y	Y	Y	Y
Determine the type and quantity of product packaging, including efforts to reduce packaging and use environmentally friendly alternatives (e.g., biodegradable packaging)	Y	Y	Y	Y	Y	Y
Determine the type of livestock and the conditions in which they are farmed		Y	Y	Y		Y
Determine the distance products travel and the type of transportation, including efforts to reduce the environmental impact of transportation		Y		Y	Y	Y

Table S3. Definitions of the Domains of Corporate Influence in the HEALTH-CORP-FB Typology [1]**.**

Domains of Corporate Influence	Definition
Governance Practices	This domain consists of activities related to how the company is managed and controlled, including actions it takes to understand and address the impacts of its activities on various stakeholders (e.g., employees, consumers, local communities, the natural environment) [2].
Political Practices	This domain consists of activities undertaken to influence government policy or processes in ways that are favourable to the commercial entity [3,4].
Preference and Perception Shaping Practices	This domain consists of activities undertaken to shape preferences for products and/or influence perceptions about products and their health-related harms [3].
Economic Practices	This domain includes corporate activities that influence the economy and the distribution of wealth within and across societies [4,5].
Employment Practices	This domain consists of activities related to the conditions under which employment is provided [4,6].
Products & Services	This domain consists of activities related to the production and sale of products and services [7].
Environmental Practices	This domain consists of corporate activities that can influence physical and/or mental health through the impact of the activity on the natural environment [4,8].

Table S4. Definitions of the SASB 'Processed Foods' subindustry, Bloomberg 'Packaged Food' subindustry, and the S&P Global 'Food Products' subindustry.

**Sustainability Accounting Standards Board (SASB) Standards**	**Definition of the 'Processed Foods' industry:** "Processed Foods industry entities process and package foods such as bread, frozen foods, snack foods, pet foods and condiments for retail consumer consumption. Typically, these products are made ready to consume, are marketed for retail consumers and can be found on food retailers' shelves. The industry is characterised by large and complex ingredient supply chains, because many entities source ingredients from around the world. Large entities operate globally, and international opportunities are driving growth."[9](p5)
**Bloomberg ESG Scores**	**Definition of the 'Packaged Food' industry:** "Packaged Food companies engage in manufacturing food products (including but not limited to snack foods, general grocery items, meat, and dairy products). They also develop packaging materials designed to preserve perishable and non-perishable food items throughout their distribution cycles."[10](p6)
**S&P Global ESG Scores**	**Definition of the 'Food Products' sub-industry:** S&P Global maps the 'Food Products' industry to the Global Industry Classification Standard (GICS) industries of 'Agricultural Products & Services' (30202010) and 'Packaged Foods & Meats' (30202030) [11]. The corresponding definitions are:<br>• ***Agricultural Products & Services:*** **"**Producers of agricultural products. Includes crop growers, owners of plantations and companies that produce and process foods but do not package and market them. Excludes companies classified in the Forest Products Sub-Industry and those that package and market the food products classified in the Packaged Foods & Meats Sub-Industry."[12]<br>• ***Packaged Foods & Meats:*** **"**Producers of packaged foods including dairy products, fruit juices, meats, poultry, fish and pet foods."[12]

Table S5. Additional Text Results (Distribution of Assigned Fields Across Domains and ESG Frameworks).

By domain, the highest number of ESG fields were mapped to Environmental Practices (419, 31%), followed by Employment Practices (392, 29%), Governance Practices (236, 18%), Political Practices (97, 7%), Preference and Perception-Shaping Practices (93, 7%), Products and Services (68, 5%), and Economic Practices (43, 3%).

The S&P Global ESG Scores contributed the highest number of assigned fields (465/1348, 35%), followed by the Bloomberg ESG Scores (462, 34%), SASB Standards (162, 12%), GRI Standards (136, 10%) and the JUST Capital Rankings (123, 9%).

Table S6. Additional Text Results (Coverage of HEALTH-CORP-FB Activities by ESG Framework).

The JUST Capital Rankings, S&P Global ESG Scores, and the GRI Standards had fields pertaining to higher numbers of HEALTH-CORP-FB activities (range across frameworks: 36-43 activities, 40-48%) than the Bloomberg ESG Scores and the SASB Standards (29 activities (33%) and 24 activities (27%), respectively). These two groups of frameworks also differ in terms of their coverage across domains. Specifically, the Bloomberg ESG Scores and SASB Standards corresponded to lower coverage of the Political Practices, Economic Practices, and Employment Practices domains compared to the other frameworks (Table 3; Figure 2; Table S2). These observed differences suggest that frameworks focused on financial materiality may cover a smaller number of practices relevant to population health.

Table S7. Heatmaps of the characteristics of the assigned ESG fields (Packaged Foods subindustry) as a function of the ESG framework.

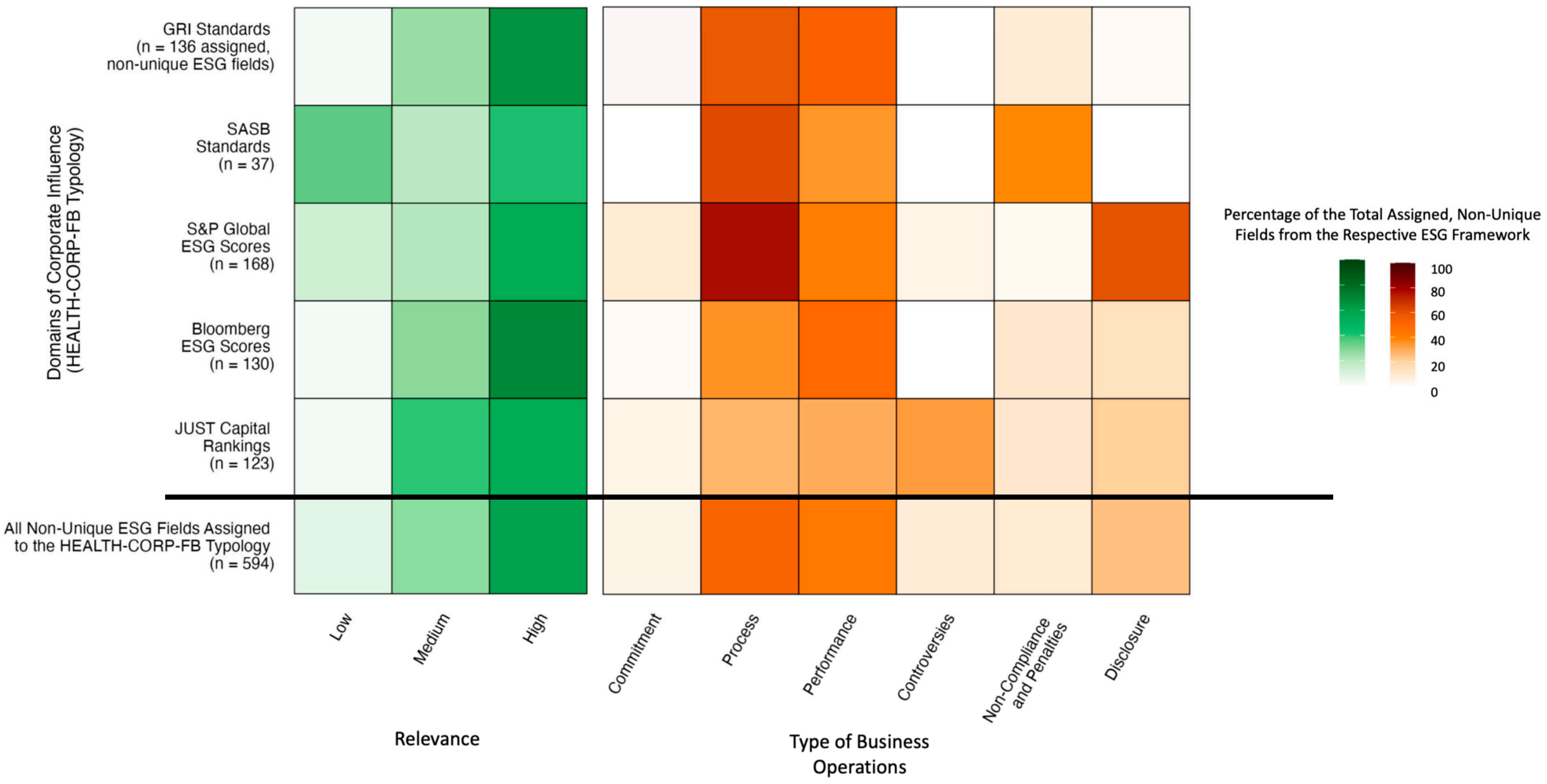

Table S8. Characteristics of the assigned ESG Fields (Packaged Foods subindustry) as a function of the ESG framework.

	Number of non-unique ESG fields corresponding to each attribute category and ESG framework (% of total assigned fields from the respective ESG framework)[a]										
	**Attribute**	*Relevance*				*Type of Business Operations*[b]					
**ESG Framework**	Total number of non-unique fields assigned from ESG framework	Low	Medium	High	N/A[c]	Commitment	Process	Performance	Controversies	Non-Compliance and Penalties	Disclosure[d]
GRI Standards	136	4 (3)	35 (26)	90 (66)	7 (5)	3 (2)	79 (58)	73 (54)	0 (0)	12 (9)	2 (1)
SASB Standards (Processed Foods)	37	13 (35)	7 (19)	15 (41)	2 (5)	0 (0)	23 (62)	13 (35)	0 (0)	14 (38)	0 (0)
S&P Global ESG Scores (Food Products)	168	22 (13)	34 (20)	94 (56)	18 (11)	15 (9)	129 (77)	69 (41)	8 (5)	4 (2)	101 (60)
Bloomberg ESG Scores (Packaged Foods)	130	3 (2)	37 (28)	90 (69)	0 (0)	2 (2)	46 (35)	66 (51)	0 (0)	13 (10)	18 (14)
JUST Capital Rankings	123	4 (3)	49 (40)	68 (55)	2 (2)	6 (5)	34 (28)	36 (29)	41 (33)	13 (11)	26 (21)

[a]Duplicative ESG fields (i.e., those assigned to more than one HEALTH-CORP-FB activity) are counted separately in this table. This is because the relevance classification is dependent on the assigned activity. However, similar distributions across domains are observed when filtered by unique ESG fields (Table S10).

[b]The counts for type of business operations do not sum to the total metric count because metrics could be classified to more than one type of business operations. For the same reason, the corresponding percentages do not sum to 100.

[c]N/A refers to not applicable. Some metrics did not receive a relevance classification because they were not assigned to a specific activity; rather, they were assigned to the domain itself.

[d]This category rarely applies to the ESG Standards as they provide the guidelines for corporate disclosures; they do not assess whether companies disclose information related to the respective topic. One field in the GRI Standards ask companies to report whether or not they publicly report certain pieces of information (e.g., results of social and environmental assessments). In the Bloomberg ESG Scores, binary fields that assess whether or not the company discloses the presence or absence of certain policies in their publicly available documents were classified as disclosure fields. However, the Bloomberg ESG scoring methodology also assesses the level of disclosure for other fields at later stages of analysis by assessing the extent of missing data.

Table S9. Additional Text Results (Characteristics of the ESG Fields as a Function of the ESG Framework).

Relevance by ESG Framework	By framework, the Bloomberg ESG Scores and the GRI Standards corresponded to the highest proportions of ESG fields that were classified as *highly relevant* to the assigned HEALTH-CORP-FB activity (Bloomberg: 90/130 fields, 69%; GRI: 90/136, 60%). The SASB Standards reflected the lowest proportion (15/37; 41%).
Type of Business Operations by ESG Framework	The highest proportion of assigned fields measuring *performance* came from the GRI Standards (73/136 fields, 54%). In addition to *process* and *performance*, the SASB Standards commonly measured *non-compliance and penalties* (14/37, 38%), the S&P Global ESG Scores commonly measured *disclosure* (101/168, 60%), and the JUST Capital Rankings commonly captured *controversies* related to the activities in the HEALTH-CORP-FB typology (41/123, 33%) (e.g., "misleading communication controversies").

Table S10. Distribution of the Type of Business Operations for Assigned ESG Fields (Packaged Food Subindustry) as a Function of the Domain of Corporate Influence [*Unique ESG Fields Only*]

	Number of UNIQUE ESG fields corresponding to attribute category and domain (% of total unique fields assigned to domain)[a,b]:						
	**Attribute**	*Type of Business Operations*[c]					
**Domain of Corporate Influence**	Total number of UNIQUE fields assigned to domain	Commitment	Process	Performance	Controversies	Non-Compliance and Penalties	Disclosure
Governance Practices	127	3 (2)	86 (68)	51 (40)	2 (2)	4 (3)	27 (21)
Political Practices	35	0 (0)	16 (46)	8 (23)	5 (14)	5 (14)	14 (40)
Preference and Perception Shaping Practices	27	1 (4)	12 (44)	6 (22)	3 (11)	11 (41)	5 (19)
Economic Practices	30	1 (3)	9 (30)	14 (47)	5 (17)	4 (13)	4 (13)
Products & Services	27	0 (0)	13 (48)	7 (26)	5 (19)	8 (30)	3 (11)
Employment Practices	117	6 (5)	60 (51)	64 (55)	7 (6)	5 (4)	33 (28)
Environmental Practices	125	6 (5)	61 (49)	82 (66)	6 (5)	3 (2)	25 (20)

[a]This table displays the counts for the unique ESG fields assigned to each domain. The ESG Fields may still repeat across domains.

[b]Relevance is not included because the relevance classification is dependent on the activity to which the ESG field is assigned. Therefore, it cannot be viewed in terms of the number of unique ESG fields.

[b]The counts do not sum to the total metric count because metrics could be classified to more than one type of business operations. For the same reason, the corresponding percentages do not sum to 100.

Table S11. Distribution of the Type of Business Operations for Assigned ESG Fields (Packaged Food Subindustry) as a Function of the ESG Framework [*Unique Metrics Only*]

	Number of UNIQUE ESG fields corresponding to each attribute category and ESG framework (% of total unique assigned fields from the respective ESG framework)[a]						
	**Attribute**	*Type of Business Operations*[b]					
**ESG Framework**	Total number of UNIQUE ESG fields assigned from ESG framework	Commitment	Process	Performance	Controversies	Non-Compliance and Penalties	Disclosure
GRI Standards	103	3 (3)	60 (58)	59 (57)	0 (0)	7 (7)	1 (1)
SASB Standards (Processed Foods)	15	0 (0)	7 (47)	8 (53)	0 (0)	4 (27)	0 (0)
S&P Global ESG Scores (Food Products)	111	8 (7)	86 (77)	52 (47)	8 (7)	3 (3)	64 (58)
Bloomberg ESG Scores (Packaged Foods)	95	2 (2)	29 (31)	58 (61)	0 (0)	5 (5)	9 (9)
JUST Capital Rankings	63	4 (6)	19 (30)	29 (46)	11 (17)	7 (11)	14 (22)

[a]Relevance is not included here because the relevance classification is dependent on the activity to which the ESG field is assigned. Therefore, it cannot be viewed in terms of the number of unique ESG fields.

[b]The counts do not sum to the total metric count because metrics could be classified to more than one type of business operations. For the same reason, the corresponding percentages do not sum to 100.

Table S12. Recommendations for disclosures/metrics that could be used to address gaps in existing ESG frameworks included in this study.[a]

HEALTH-CORP-FB Activity (Domain of Corporate Influence)	ESG frameworks investigated in this study that address this activity	Proposed Disclosure or Metric and Source (if applicable)	Importance to Population Health & Additional Notes
Exploit the use of revolving doors (i.e., employees who move between positions in industry and government) (political practices)	None	Number and percentage of company-affiliated individuals who have worked in government agencies within the last ten years, with respect to:<br>a) Senior management employees (i.e., employees that are "at most two management levels from the CEO"[13])<br>b) Internal or external lobbyists hired by the firm	The movement of public officials to the private sector is regulated in many regions (e.g., 'cooling-off periods') [14]. This type of movement is believed to have a corrupting effect on the implementation of public policy [14,15]. In the United States, OpenSecrets maintains a database of individuals who have moved between the public and private sectors [16].
Take or threaten legal action in response to unfavourable policies (political practices)	None	Litigation the company, its subsidiaries, and/or those acting on behalf of the company (e.g., trade associations) have engaged in that pertain to proposed public health measures in the last three years, including:<br>a) The case name (e.g., American Beverage Association v. City & County of San Francisco)<br>b) The public health measure related to the case (e.g., health warnings on advertisements for sugary drinks)<br>c) Details of the case (e.g., the ABA claimed that the city of San Francisco violated the First Amendment and the Due Process Clause of the Fourteenth Amendment to the United States Constitution)<br>d) Current status of the case (e.g., Ninth Court of Appeals ruled in favour of the ABA[17])<br>(adapted from the Handbook for SDG-Aligned Food Companies[18])	Litigation is a strategy used by some F&B companies to block the implementation of policies designed to protect population health [19–21].<br><br>This metric would not include threats of litigation intended to deter public health action.<br><br>In some countries such as the United States, companies are required to disclose any 'material' legal proceedings (i.e., relevant to investor decision making) in their financial reports [22].
Engage in lobbying, including via third parties (political practices)	JUST Capital (disclosure metric), S&P Global ESG Scores (performance, process, disclosure metric)	Information about lobbying activities that the company, its subsidiaries, and/or those acting on behalf of the company (e.g. trade associations) have engaged in during the past year, including information on:<br>a) Annual financial or in-kind contributions for lobbying activities, including internal and external expenses and costs of membership to lobbying associations (broken down by the region in which the lobbying took place),	Lobbying is a strategy that can be undertaken by F&B companies to undermine, delay, or block the implementation of policies designed to protect population health (e.g., taxation on harmful products, front-of-pack labelling regulations) [19–21,24,25].

		b) the main topics covered by the commercial entity's (or relevant affiliate's) lobbying activities (e.g., proposed taxation on harmful products), and<br>a) the commercial entity's (or relevant affiliate's) primary position(s) in relation to the respective topics (e.g., supportive, oppositional)<br>(adapted from Disclosure Requirement G1-5 from the European Sustainability Reporting Standards)[23]	
Develop and/or use features of product packaging (e.g., toys, colours) that are appealing to children (preference and perception shaping practices)	None	Regarding responsible use of promotional toys, games, vouchers and competitions; does the company commit to:<br>• Never make use of promotional games, toys, vouchers, competitions etc. in their marketing to children and/or teens<br>• Using promotional games, toys, vouchers, competitions etc. only in accordance to WHO regional standards<br>• Using promotional games, toys, vouchers, competitions etc. only in accordance to the company's own or industry association-related standards for marketing to children and/or teens<br>• No commitment<br>• No information<br>(metric from the 2021 Access to Nutrition Index)[26]	The World Health Organization has recently released updated guidelines on regulations to limit marketing of harmful food and beverage products to children. Based on their review of the evidence, the WHO concluded that "Children continue to be exposed to powerful marketing of HFSS foods [high in saturated fatty acids, trans-fatty acids, free sugars and/or salt, usually highly processed], consumption of which is<br>associated with negative health effects (8, 9). Such marketing is prevalent…<br>and uses many techniques appealing to<br>young audiences (9) [e.g., promotional characters, toys]."[27]
Determine the level of processing of products (products & services)	JUST Capital (indirect measure of public sentiment towards processed foods and associated revenue from processed foods)[28]	Amount and percentage of annual revenue generated from food and beverage products classified as 'ultra-processed' using the Nova classification system, net change over the past five years, and any associated commitments/targets related to reducing the proportion of ultra-processed foods in the company's product portfolio (adapted from the 'Handbook for SDG-Aligned Food Companies'[18])	There is significant evidence indicating that ultra-processed foods have important implications for population health that extend beyond their nutritional value.[29–32] For example, a recent umbrella review of epidemiological meta-analyses found class 1 (i.e., convincing) evidence that exposure to ultra-processed foods is associated with higher risks of incident cardiovascular disease related mortality, type 2 diabetes, and prevalent anxiety outcomes.[30] The Nova classification, groups F&B products into four main categories of processing (unprocessed, processed culinary ingredients, processed foods, and ultra-processed foods) [33].

Determine the portion size of products (products and services)	None	In the last 3 years, has the company invested in or developed products with smaller packaging sizes or serving sizes, aimed to improve consumer portion control?<br>• Yes, across multiple products or product categories<br>• Yes, limited to one type of product or product category<br>• No<br>• No information<br>• Not applicable<br>(metric from the 2021 Access to Nutrition Index)[26]	Portion size has been associated with higher energy intake, with implications for the obesity crisis [34,35].
Determine the extent of support for breastfeeding in the workplace (employment practices)	None	a) Existence of the following types of support for breastfeeding employees:<br>i. Lactation rooms<br>ii. Fridges to store expressed breastmilk<br>iii. Regular breaks to express breastmilk<br>iv. Flexible working arrangements to support breastfeeding<br>b) Number and percentage of facilities in which each of these types of supports are offered<br>(adapted from the 2021 Access to Nutrition Index)[26]	Breastfeeding is associated with significant health benefits for both mother and child (e.g., reduced risk of some maternal cancers, reduced risk of obesity later in life for the breastfed individual) [36–38]. Lack of support for breastfeeding in the workplace has been identified as a key barrier [39,40].

[a]For additional recommendations of ESG metrics focused on nutrition, please see O'Hearn and colleagues [41]. The "Handbook for SDG-Aligned Food Companies" produced by the Columbia Center on Sustainable Development also provides recommendations for metrics that F&B companies could use to track and disclose their performance with respect to the Sustainable Development Goals (including metrics related to human health) [18]. Finally, Chan and colleagues offer a set of 41 reporting metrics that they designed for food companies operating in Australia in the areas of: corporate strategy and governance, product formulation, nutrition labelling and information, promotion practices, and product accessibility and affordability [42].

References

1. Burgess, R.; Freudenberg, N.; Grierson, L.; Srebotnjak, T.; Ransome, Y. Food & Beverage Industry Activities That Influence Population Health: Development of the HEALTH-CORP-FB Typology. *Int. J. Soc. Determ. Health Health Services*.
2. Corporate Governance Definition: How It Works, Principles, and Examples Available online: https://www.investopedia.com/terms/c/corporategovernance.asp (accessed on 1 September 2023).
3. Madureira Lima, J.; Galea, S. Corporate Practices and Health: A Framework and Mechanisms. *Globalization and Health* **2018**, *14*, 1–12. https://doi.org/10.1186/s12992-018-0336-y.
4. Gilmore, A.B.; Fabbri, A.; Baum, F.; Bertscher, A.; Bondy, K.; Chang, H.-J.; Demaio, S.; Erzse, A.; Freudenberg, N.; Friel, S.; et al. Defining and Conceptualising the Commercial Determinants of Health. *Lancet* **2023**, *401*, 1194–1213. https://doi.org/10.1016/S0140-6736(23)00013-2.
5. Baum, F.E.; Sanders, D.M.; Fisher, M.; Anaf, J.; Freudenberg, N.; Friel, S.; Labonté, R.; London, L.; Monteiro, C.; Scott-Samuel, A.; et al. Assessing the Health Impact of Transnational Corporations: Its Importance and a Framework. *Globalization and Health* **2016**, *12*, 1–7. https://doi.org/10.1186/s12992-016-0164-x.
6. Occupational Health. Available online: https://www.ilo.org/safework/areasofwork/occupational-health/lang--en/index.htm (accessed on 1 March 2023).
7. Knai, C.; Petticrew, M.; Capewell, S.; Cassidy, R.; Collin, J.; Cummins, S.; Eastmure, E.; Fafard, P.; Fitzgerald, N.; Gilmore, A.B.; et al. The Case for Developing a Cohesive Systems Approach to Research across Unhealthy Commodity Industries. *BMJ global health* **2021**, *6*. https://dx.doi.org/10.1136/bmjgh-2020-003543.
8. Sattler, B. Environmental Health. *Policy, Politics, & Nursing Practice* **2003**, *4*, 4–5. https://doi.org/10.1177/1527154402239448.
9. *Processed Foods Sustainability Accounting Standard*. (2023, June). SASB Standards. https://sasb.ifrs.org/standards/download/ (Accessed on 27 September 2023)
10. Environmental and Social Scores: Methodology Industry Guide - Packaged Food and Beverages 2021. (accessed on 15 November 2023).
11. S&P Global CSA Industry-GICS Sub-Industry Mapping 2023. Available online: https://www.spglobal.com/spdji/en/documents/index-policies/sp-global-csa-industry-gics-sub-industry-mapping.pdf (accessed on 22 November 2023).
12. GICS Structure 2023. Available online: https://www.spglobal.com/spdji/en/landing/topic/gics/ (accessed 22 November 2023).
13. S&P Global Corporate Sustainability Assessment Food Products Questionnaire and Rationale 2021. (accessed 22 November 2023).
14. Martini, M. *Cooling-Off Periods: Regulating the Revolving Door*; Transparency International: Berlin, Germany, 2015.
15. McKay, A.M.; Lazarus, J. Policy Consequences of Revolving-Door Lobbying. *Political Research Quarterly* **2023**, *76*, 1780–1793. https://doi.org/10.1177/10659129231177648.
16. Overview: Revolving Doors Available online: https://www.opensecrets.org/revolving-door (accessed on 6 October 2024).
17. American Beverage Association v. City and County of San Francisco (2016) Available online: https://www.publichealthlawcenter.org/litigation-tracker/american-beverage-association-et-al-v-city-and-county-san-francisco-2016 (accessed on 6 October 2024).
18. Handbook for SDG-Aligned Food Companies 2021. Available online: https://ccsi.columbia.edu/sites/default/files/content/docs/19%20CCSI%20Four%20pillars%20full%20report%20rhr.pdf. (accessed 22 Nov 2023).
19. Roache, S.A.; Platkin, C.; Gostin, L.O.; Kaplan, C. Big Food and Soda versus Public Health: Industry Litigation against Local Government Regulations to Promote Healthy Diets. *Fordham Urb. L.J.* **2017**, *45*, 1051–1090.

20. Mialon, M.; Swinburn, B.; Sacks, G. A Proposed Approach to Systematically Identify and Monitor the Corporate Political Activity of the Food Industry with Respect to Public Health Using Publicly Available Information. Obes. Rev. Off. J. Int. Assoc. Study Obes. 2015, 16, 519–530
21. Ulucanlar, S.; Lauber, K.; Fabbri, A.; Hawkins, B.; Mialon, M.; Hancock, L.; Tangcharoensathien, V.; Gilmore, A.B. Corporate Political Activity: Taxonomies and Model of Corporate Influence on Public Policy. *Int. J. Health Policy Manag.* **2023**, *12*, 1–22. https://doi.org/10.34172/ijhpm.2023.7292.
22. 17 CFR § 229.103 - (Item 103) Legal Proceedings. Available online: https://www.law.cornell.edu/cfr/text/17/229.103 (accessed on 6 October 2024).
23. European Commission. Commission Delegated Regulation (EU) 2023/2772 of 31 July 2023 Supplementing Directive 2013/34/EU of the European Parliament and of the Council as Regards Sustainability Reporting Standards; European Commission: Brussels, Belgium, 2023.
24. Mialon, M.; Charry, D.A.G.; Cediel, G.; Crosbie, E.; Scagliusi, F.B.; Tamayo, E.M.P. "I Had Never Seen so Many Lobbyists": Food Industry Political Practices during the Development of a New Nutrition Front-of-Pack Labelling System in Colombia. *Public Health Nutr.* **2021**, *24*, 2737–2745. https://doi.org/10.1017/S1368980020002268.
25. Mialon, M.; Corvalan, C.; Cediel, G.; Scagliusi, F.B.; Reyes, M. Food Industry Political Practices in Chile: "The Economy Has Always Been the Main Concern." *Globalization and Health* **2020**, *16*. https://doi.org/10.1186/s12992-020-00638-4.
26. Access to Nutrition Initiative *Global Access to Nutrition Index 2021: Methodology*; Access to Nutrition Initiative, 2020; Available online: https://accesstonutrition.org/app/uploads/2020/06/Global-Index-2021-Methodology-FINAL.pdf (accessed 22 Oct 2023).
27. *Policies to Protect Children from the Harmful Impact of Food Marketing: WHO Guideline*; World Health Organization: Geneva, 2023; Available online: https://www.who.int/publications/i/item/9789240075412 (accessed 22 Oct 2023).
28. *JUST Capital 2023 Ranking Methodology*; JUST Capital Foundation, Inc.: New York, NY, USA, 2023.
29. Chang, K.; Gunter, M.J.; Rauber, F.; Levy, R.B.; Huybrechts, I.; Kliemann, N.; Millett, C.; Vamos, E.P. Ultra-Processed Food Consumption, Cancer Risk and Cancer Mortality: A Large-Scale Prospective Analysis within the UK Biobank. *eClinicalMedicine* **2023**, *56*. https://doi.org/10.1016/j.eclinm.2023.101840.
30. Lane, M.M.; Gamage, E.; Du, S.; Ashtree, D.N.; McGuinness, A.J.; Gauci, S.; Baker, P.; Lawrence, M.; Rebholz, C.M.; Srour, B.; et al. Ultra-Processed Food Exposure and Adverse Health Outcomes: An Umbrella Review of Epidemiological Meta-Analyses 2023, *384*, e077310. https://doi.org/10.1136/bmj-2023-077310
31. Monteiro, C.A.; Martínez-Steele, E.; Cannon, G. Reasons to Avoid Ultra-Processed Foods. *BMJ* **2024**, *384*, q439. https://doi.org/10.1136/bmj.q439.
32. Pagliai, G.; Dinu, M.; Madarena, M.P.; Bonaccio, M.; Iacoviello, L.; Sofi, F. Consumption of Ultra-Processed Foods and Health Status: A Systematic Review and Meta-Analysis. *British Journal of Nutrition* **2021**, *125*, 308–318.
33. Martinez-Steele, E.; Khandpur, N.; Batis, C.; Bes-Rastrollo, M.; Bonaccio, M.; Cediel, G.; Huybrechts, I.; Juul, F.; Levy, R.B.; da Costa Louzada, M.L.; et al. Best Practices for Applying the Nova Food Classification System. *Nat. Food* **2023**, *4*, 445–448. https://doi.org/10.1038/s43016-023-00779-w.
34. Higgins, K.A.; Hudson, J.L.; Hayes, A.M.R.; Braun, E.; Cheon, E.; Couture, S.C.; Gunaratna, N.S.; Hill, E.R.; Hunter, S.R.; McGowan, B.S.; et al. Systematic Review and Meta-Analysis on the Effect of Portion Size and Ingestive Frequency on Energy Intake and Body Weight among Adults in Randomized Controlled Feeding Trials. *Advances in Nutrition* **2022**, *13*, 248–268. https://doi.org/10.1093/advances/nmab112.
35. Livingstone, M.B.E.; Pourshahidi, L.K. Portion Size and Obesity. *Advances in Nutrition* **2014**, *5*, 829–834. https://doi.org/10.3945/an.114.007104.
36. Chowdhury, R.; Sinha, B.; Sankar, M.J.; Taneja, S.; Bhandari, N.; Rollins, N.; Bahl, R.; Martines, J. Breastfeeding and Maternal Health Outcomes: A Systematic Review and Meta-Analysis. *Acta Paediatrica* **2015**, *104*, 96–113. https://doi.org/10.1111/APA.13102.

37. Pérez-Escamilla, R.; Tomori, C.; Hernández-Cordero, S.; Baker, P.; Barros, A.J.D.; Bégin, F.; Chapman, D.J.; Grummer-Strawn, L.M.; McCoy, D.; Menon, P.; et al. Breastfeeding: Crucially Important, but Increasingly Challenged in a Market-Driven World. *The Lancet* **2023**, *401*, 472–485. https://doi.org/10.1016/S0140-6736(22)01932-8.
38. Horta, B.L.; Rollins, N.; Dias, M.S.; Garcez, V.; Pérez-Escamilla, R. Systematic Review and Meta-Analysis of Breastfeeding and Later Overweight or Obesity Expands on Previous Study for World Health Organization. *Acta Paediatrica* **2023**, *112*, 34–41. https://doi.org/10.1111/apa.16460.
39. Tomori, C.; Hernández-Cordero, S.; Busath, N.; Menon, P.; Pérez-Escamilla, R. What Works to Protect, Promote and Support Breastfeeding on a Large Scale: A Review of Reviews. *Maternal & Child Nutrition* **2022**, *18*, e13344. https://doi.org/10.1111/mcn.13344.
40. Litwan, K.; Tran, V.; Nyhan, K.; Pérez-Escamilla, R. How Do Breastfeeding Workplace Interventions Work?: A Realist Review. *Int J Equity Health* **2021**, *20*, 148. https://doi.org/10.1186/s12939-021-01490-7.
41. O'Hearn, M.; Gerber, S.; Cruz, S.M.; Mozaffarian, D. The Time Is Ripe for ESG + Nutrition: Evidence-Based Nutrition Metrics for Environmental, Social, and Governance (ESG) Investing. *Eur. J. Clin. Nutr.* **2022**, *76*, 1047–1052. https://doi.org/10.1038/s41430-022-01075-9.
42. Chan, J.; Robinson, E.; Mhurchu, C.N.; Sacks, G. Proposed Best-Practice Nutrition-Related Corporate Reporting Metrics for Australian Food Manufacturers, Quick-Service Restaurants and Retailers. *Public Health Nutr.* **2025**, *28*, 1–33. https://doi.org/10.1017/S1368980025101134.
